# Anorexia-Induced Hypoleptinemia Drives Adaptations in the JAK2/STAT3 Pathway in the Ventral and Dorsal Hippocampus of Female Rats

**DOI:** 10.3390/nu16081171

**Published:** 2024-04-15

**Authors:** Giorgia Targa, Francesca Mottarlini, Beatrice Rizzi, Sofia Taddini, Susanna Parolaro, Fabio Fumagalli, Lucia Caffino

**Affiliations:** 1 Department of Pharmacological and Biomolecular Sciences ‘Rodolfo Paoletti’, Università degli Studi di Milano, Via Balzaretti 9, 20133 Milano, Italy; giorgia.targa@unimi.it (G.T.); francesca.mottarlini@guest.unimi.it (F.M.); beatrice.rizzi@unimi.it (B.R.); sofia.taddini@unimi.it (S.T.); susanna.parolaro@unimi.it (S.P.); fabio.fumagalli@unimi.it (F.F.); 2 Center for Neuroscience, University of Camerino, 62032 Camerino, Italy

**Keywords:** anorexia nervosa, activity-based anorexia, leptin, hippocampus

## Abstract

Leptin is an appetite-regulating adipokine that is reduced in patients with anorexia nervosa (AN), a psychiatric disorder characterized by self-imposed starvation, and has been linked to hyperactivity, a hallmark of AN. However, it remains unknown how leptin receptor (LepR) and its JAK2-STAT3 downstream pathway in extrahypothalamic brain areas, such as the dorsal (dHip) and ventral (vHip) hippocampus, crucial for spatial memory and emotion regulation, may contribute to the maintenance of AN behaviors. Taking advantage of the activity-based anorexia (ABA) model (i.e., the combination of food restriction and physical activity), we observed reduced leptin plasma levels in adolescent female ABA rats at the acute phase of the disorder [post-natal day (PND) 42], while the levels increased over control levels following a 7-day recovery period (PND49). The analysis of the intracellular leptin pathway revealed that ABA rats showed an overall decrease of the LepR/JAK2/STAT3 signaling in dHip at both time points, while in vHip we observed a transition from hypo- (PND42) to hyperactivation (PND49) of the pathway. These changes might add knowledge on starvation-induced fluctuations in leptin levels and in hippocampal leptin signaling as initial drivers of the transition from adaptative mechanisms to starvation toward the maintenance of aberrant behaviors typical of AN patients, such as perpetuating restraint over eating.

## 1. Introduction

Leptin is an adipokine mediator of adipose–nerve tissue communication and plays a crucial role in regulating appetite, feeding, body mass and energy expenditure [1]. It is produced from adipocytes, circulates in the blood [2] and crosses the blood–brain barrier to modulate the anorexigenic response via the leptin receptor (LepR) located in neuropeptide Y/agouti-related protein (NPY/AgRP) and pro-opiomelanocortin (POMC) neurons in the hypothalamus [3,4,5]. Upon binding to its receptor, leptin can exert its effects by activating the Janus kinase 2/signal transducer and activator of the transcription 3 (JAK2/STAT3) signaling pathway [6]. Following STAT3 phosphorylation and translocation from the cytoplasm into the nucleus, it regulates the transcription of numerous target genes contributing to the control of energy homeostasis [7,8]. LepRs are also highly expressed in extra-hypothalamic brain regions [9], such as the hippocampus [10], where they modulate synaptic plasticity, learning and memory performances [11] and depression-related behavior [12,13].

During the condition of being underweight, hypoleptinemia represents a key marker of starvation and LepR signaling, together with other secretory feeding-induced hormones, it mediates physiological and behavioral adaptations to starvation, such as reduction in energy expenditure, body temperature and reproductive behaviors [14]. While intermittent starvation is known to improve body performance by acting as an acute stressor, a prolonged period of starvation instead acts as a chronic stressor that might endanger survival via maladaptive consequences, such as increasing energy expenditure to foraging and risky behaviors and thus, in turn, reducing predation avoidance, increasing aggressive interactions and reducing reproductive behavior [15]. In this scenario, the major cause of voluntary weight and fat loss resulting in severe emaciation is anorexia nervosa (AN), a psychiatric disorder that leads to pronounced hypoleptinemia. AN mainly affects adolescent females [16] and it is characterized by severe emaciation caused by obsessive control over weight, excessive physical activity and cognitive inflexibility [17,18]. In underweight AN patients, leptin plasma and cerebrospinal fluid levels are markedly reduced and positively correlated to the patients’ body mass index and body fat mass [19,20,21,22]. It has been postulated that alterations in leptin levels, together with other appetite modulators, are the primary consequences of the nutritional changes occurring with AN. However, even though most of those alterations disappear following bodyweight recovery [23], our hypothesis is that they may contribute to the maintenance of both aberrant eating behaviors and/or other comorbid features, such as cognitive impairment, anxiety- and depressive-like behaviors. In fact, hypoleptinemia, an index of AN disorder severity [17,18,24], regulates the neuroendocrine system inducing physiological and endocrinological changes that first drive the adaptation process required to enhance survival while, later on, it contributes to exacerbating bodyweight loss [14]. During refeeding, leptin levels progressively increase along with bodyweight recovering and, when weight restoration is rapid, leptin reaches values disproportionately higher than normal [19,20,21,25].

Interestingly, it has been demonstrated that starvation-induced hypoleptinemia regulates not only energy homeostasis but also the hedonic and motivational aspects of reward [26], affective state and cognitive functions [27]. Moreover, it has been proposed that hypoleptinemia could play a role in the elevated physical activity typical of women with AN [28]. Off-label treatment of AN patients with metreleptin, a recombinant analog of human leptin, entails an increased motivation to overcome their eating disorder, a reduction in hyperactivity and an improvement in depressive-like symptomatology and cognitive impairment [29,30,31]. Accordingly, in rodent studies, leptin treatment via minipumps or intracerebroventricular administration after the initiation of food restriction reduced running activity on a wheel in the starvation-induced hyperactivity and in the activity-based anorexia (ABA) rat models, thus allowing an association between hypoleptinemia and hyperactivity [32,33,34].

In this scenario, our hypothesis is that AN-induced alterations in the crosstalk among peripheral and central regulators of energy balance, such as leptin, may represent not only homeostatic adaptations to malnutrition, but also contribute to the development and/or the maintenance of maladaptive behaviors typical of the disorder. On this basis, we expect that low circulating leptin levels might initially trigger a reorganization of the leptin-mediated pathway in extra-hypothalamic brain areas, which, even following regaining of bodyweight and consequent fluctuation of leptin levels, persistently contributes to sustain aberrant behaviors in AN. Since little is known about the molecular mechanisms underlying the modulation of LepR signaling in emotional- and cognitive-related brain areas in a condition of AN-induced starvation, the aim of our work was to investigate the impact of the anorexic phenotype on the LepR/JAK2/STAT3 pathway in the ventral and dorsal hippocampi (vHip and dHip, respectively). To this purpose, female adolescent rats have been subjected to the ABA paradigm, the gold standard model of AN that combines minimal food intake with intense physical activity [35] and sacrificed at two different time points, i.e., in the acute phase of the phenotype (i.e., 25% of weight loss paralleled by an exponential increase in running activity on the wheel) and after a 7-day period of bodyweight recovery. We compared the ABA-induced effects to the consequences of caloric restriction or to running wheel activity alone to dissect their specific contribution to the maintenance of the ABA phenotype. At a molecular level, in addition to the analysis of the LepR expression, we evaluated the expression and phosphorylation levels of JAK2 and STAT3, as indexes of activation levels of the LepR pathway. Moreover, we evaluated the gene expression of two STAT3-dependent genes, *Socs3* and *Dnmt1*, as an index of STAT3 activation. In particular, Socs3, a cytokine signaling suppressor, exerts its function by controlling the negative feedback on the LepR signaling via JAK2/STAT3 pathway inhibition [36]. The DNA methyl transferase *Dnmt1*, through DNA methylation, inhibits the binding of transcription factors or indirectly blocks the chromatin remodeling functions by repressing the hippocampal transcription of genes involved in LTP and memory consolidation [37,38]. In this manuscript, we employed animals from a previously published study [39] for two primary reasons. Firstly, we aimed to comply with the new regulations regarding the use of animals for scientific research, which seek to minimize their number, where possible. Secondly, we wanted to utilize the brain areas of animals whose behavioral characterization of the anorexic phenotype was already known. In particular, in the acute phase of the pathology, we observed that ABA rats reduced bodyweight significantly more than only food-restricted rats, despite eating the same amount of food. Concurrently, unlike EXE animals that maintained a stable activity on the wheel, ABA rats constantly increased running activity over days [39]. The generation of this food restriction-induced hyperactivity represents a hallmark of the maladaptive behavior characteristic of AN [40].

## 2. Materials and Methods

### 2.1. Animals and Housing

The experimental paradigm was performed on adolescent female Sprague–Dawley rats (Charles River, Calco, Italy) at the Department of Pharmacological and Biomolecular Sciences at the University of Milan. All animal procedures were conducted following the principles set out in the subsequent laws and policies governing the care and use of laboratory animals: Italian Governing Law (D.lgs 26/2014; Authorization n.19/2008—A issued 6 March 2008, by Ministry of Health); the NIH Guide for the Care and Use of Laboratory Animals (2011 edition) and EU directives and guidelines (EEC Council Directive 2010/63/UE). Authorization for animal use has been obtained from the Italian Ministry of Health (#898-2016-PR and 833-2019-PR). All efforts were made to reduce animal suffering and to use the lowest number of animals: for ethical reasons, ABA rats were not allowed to lose more than 25% of their initial bodyweight. The experiments have been reported in observance of the ARRIVE guidelines.

Upon their arrival, rats were maintained under standard conditions of temperature (21 ± 1 °C) and humidity (50–60%) and with a reversed 12 h light/dark cycle (lights on: 10.30 p.m.; lights off: 10.30 a.m.). Animals were nourished with standard rat chow (ssniff Spezialdiäten GmbH, Soest, Germany) and with tap water *ad libitum*. To avoid the so-called “litter effects”, only up to two female siblings were taken from each litter [41].

### 2.2. Experimental Design

Adolescent female rats arrived on postnatal day (PND) 28, housed in groups of four in standard home cages and left undisturbed to get accustomed to the reversed light/dark cycle until PND31, when rats started to be weighed daily. At PND35, animals were individually housed; however, the cages were placed next to each other to provide sight, acoustic and odor contact. Animals were arbitrarily divided into four groups, assuring to place siblings, if present, in different experimental groups: control (CTRL) group, housed in standard homecages with food *ad libitum*; food-restricted (FR) group, housed in standard homecages with food limited for 2 h/day; exercise (EXE) group, allowed to freely access a mechanical wheel (activity wheel BIO-ACTIVW- R cage, Bioseb, Vitrolles, France) to experience voluntary running activity with food *ad libitum*; activity-based anorexia (ABA) group, allowed to freely access a mechanical wheel to experience voluntary running activity with food limited for 2 h/day. 

**Figure 1:**
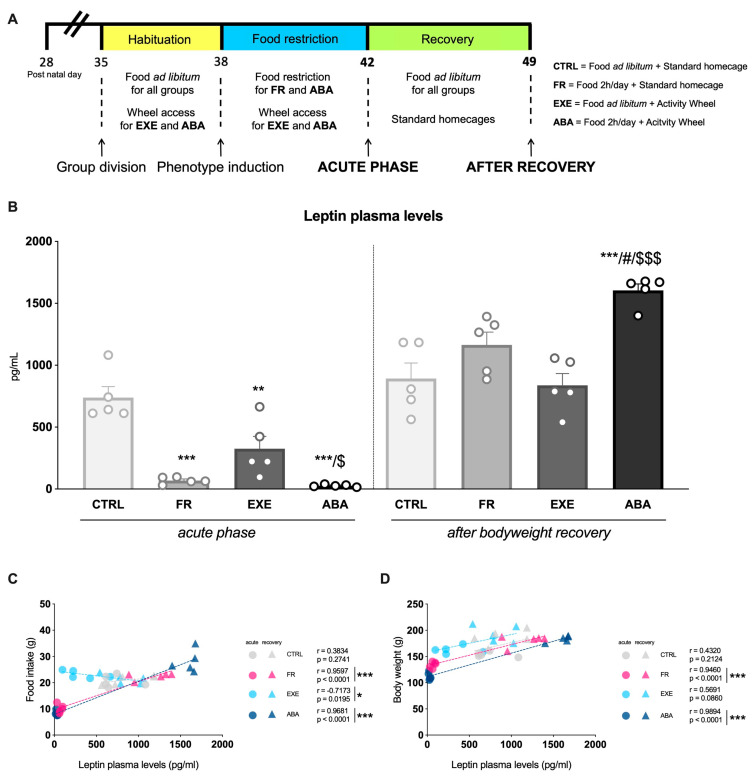
**Effect of the ABA paradigm induction on the circulating leptin levels.** Panel (**A**) shows the timeline of the experimental paradigm. Panel (**B**) represents the levels of circulating leptin measured in CTRL, FR, EXE and ABA rats in the acute phase (PND42) of the phenotype and after a period of bodyweight recovery (PND49). Data are expressed in scatter plot bar graphs as pg/mL and represent the mean ± SEM of five rats per group. Two-way ANOVA followed by Tukey’s multiple comparison test ** *p* < 0.01, *** *p* < 0.001 vs. CTRL; # *p* < 0.05 vs. FR; $ *p* < 0.05, $$$ *p* < 0.001 vs. EXE. Pearson’s product–moment correlation (r) analyses between leptin plasma levels and food intake (g) (panel **C**) or bodyweight (g) (panel **D**) are represented as group mean for CTRL, FR, EXE and ABA rats at both PND42 and PND49. * *p* < 0.05, *** *p* < 0.001. CTRL: control; FR: food-restricted; EXE: exercise; ABA: activity-based anorexia.

After 3 days of familiarization with the new environment, at PND38, the food restriction started and FR and ABA rats underwent caloric restriction with full and free access to food for only 2 h/day from 10.30 to 12.30 a.m. This extended feeding period, compared to the original protocol that dictates 1 h–1.5 h/day of food availability, was made necessary to reduce mortality since younger and lighter rats show greater vulnerability to ABA [42]. Of note, during the 2 h of food access, wheels were blocked to prevent rats from favoring running rather than eating [43]. At PND42, ABA animals reached the anorexic phenotype, thus showing a bodyweight loss equivalent to 25% compared to the CTRL physiological bodyweight increment in parallel to an exponential increment in wheel activity [44]. Half of the rodents were sacrificed by decapitation at PND42 (acute phase), while the remaining animals were transferred into regular housing cages with food *ad libitum* (from PND42 to PND49) to allow bodyweight recovery and then sacrificed (PND49). A representative timeline of the experiment is shown in Figure 1A. 

vHip and dHip were immediately dissected from the whole brain according to the plates 47–90 (from −1.72 mm to −6.84 mm from Bregma) of the Paxinos and Watson Rat Brain Atlas [45], frozen on dry ice and stored at −80 °C, and plasma were collected in a previous study [39]. Bodyweight, food intake and wheel-running activity were evaluated per each animal daily at 9.30 a.m., and these behavioral measurements were already reported in our previous study [39].

### 2.3. Plasma Collection and ELISA Assay

After decapitation, trunk blood was immediately collected in vials containing EDTA (0.5 M, pH 8) and centrifuged at 3000× *g* for 20 min. The supernatant, corresponding to the plasma fraction, was isolated and stored at −80 °C until the analysis. According to the manufacturer’s instructions, leptin plasma levels were assessed by ELISA assay using a commercial kit (R&D systems, Biotechne, Milan, Italy, RRID: AB_2943468). 

### 2.4. Post-Synaptic Density Isolation and Western Blot Analyses

Proteins from vHip and dHip were extracted as previously reported with minor modifications [46]. Briefly, tissues were homogenized and an aliquot of each homogenate, the nuclear fraction, the cytosolic fraction and the Triton X-100 insoluble fraction (TIF, post-synaptic density) were stored at −20 °C.

The total amount of proteins in the different fraction was quantified by means of the Bradford Protein Assay procedures (Bio-Rad, Milan, Italy). Equal amounts of proteins of the nuclear fraction (15 μg), cytosolic fraction (15 μg) and TIF fraction (10 μg) were loaded and run on a sodium dodecyl sulfate 8% polyacrylamide gel and then electrophoretically transferred onto nitrocellulose membranes (GE Healthcare, Milan, Italy). The entire nitrocellulose blot was cut close to the molecular weight at which protein bands are expected to be detected, as indicated by their specific molecular weight and the information depicted in the antibody datasheet. Blot strips were then blocked for 1 h at room temperature with I-Block solution (Life Technologies Italia, Segrate, Italy) in TBS 0.1% Tween-20 buffer and incubated with antibodies against the phosphorylated forms of the proteins and then stripped and re-probed with the antibodies against corresponding total proteins. The conditions of the primary antibodies were the following: anti-Leptin Receptor (1:1000, Biotechne, cod: NB100-40796, RRID: AB_715008), anti-pJAK2_Tyr1007/1008_ (1:1000, Cell signaling technology, Milan, Italy, cod: 3776, RRID: AB_2617123), anti-JAK2 (1:1000, Cell signaling technology, cod: 3230, RRID: AB_2128522), anti-pSTAT3_Tyr705_ (1:1000, Cell signaling technology, cod: 9131, RRID: AB_331586), anti-STAT3 (1:1000, Cell signaling technology, cod: 4904, RRID: AB_331269). Results were standardized to β-actin control protein (1:7500, Sigma Aldrich, Milan, Italy, cod: A5441, RRID: AB_476744) detected at 43 kDa.

**Figure 2:**
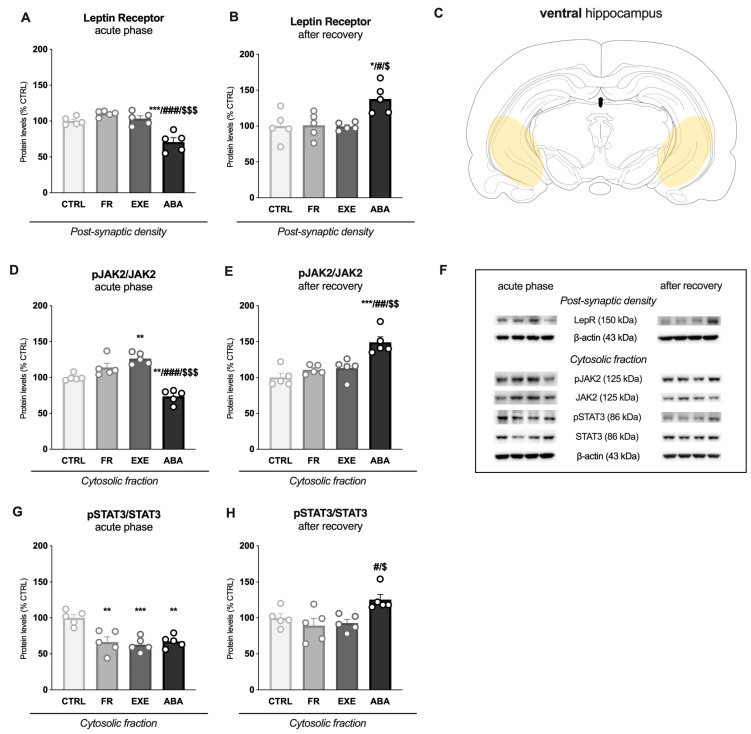
**Effects of the ABA paradigm induction on the leptin receptor and signaling in the ventral hippocampus.** LepR levels, measured in the PSD, are shown in the acute phase of the pathology (**A**) and after a 7-day recovery period (**B**). Levels of phosphorylation and total protein expression of JAK2 represented as a ratio of phospho(p)-JAK2 in Tyr1007/1008 over total JAK2 and levels of phosphorylation and total protein expression of STAT3 represented as a ratio of phospho(p)-STAT3 in Tyr705 over total STAT3 were measured in the cytosolic fraction in the acute phase of the phenotype (**D**,**G**) and after a period of bodyweight recovery (**E**,**H**). Data are expressed in scatter plot bar graphs as % of CTRL animals and represent the mean ± SEM of five rats per group. Representative immunoblots for each protein are shown in panel (**F**). Panel (**C**) shows the representative image of the subregion of interest (vHip: ventral hippocampus). * *p* < 0.05, ** *p* < 0.01, *** *p* < 0.01 vs. CTRL; # *p* < 0.05, ## *p* < 0.01, ### *p* < 0.001 vs. FR; $ *p* < 0.05, $$ *p* < 0.01, $$$ *p* < 0.001 vs. EXE. (Two-way ANOVA followed by Tukey’s multiple comparisons test). CTRL: control; FR: food-restricted; EXE: exercise; ABA: activity-based anorexia.

**Figure 3:**
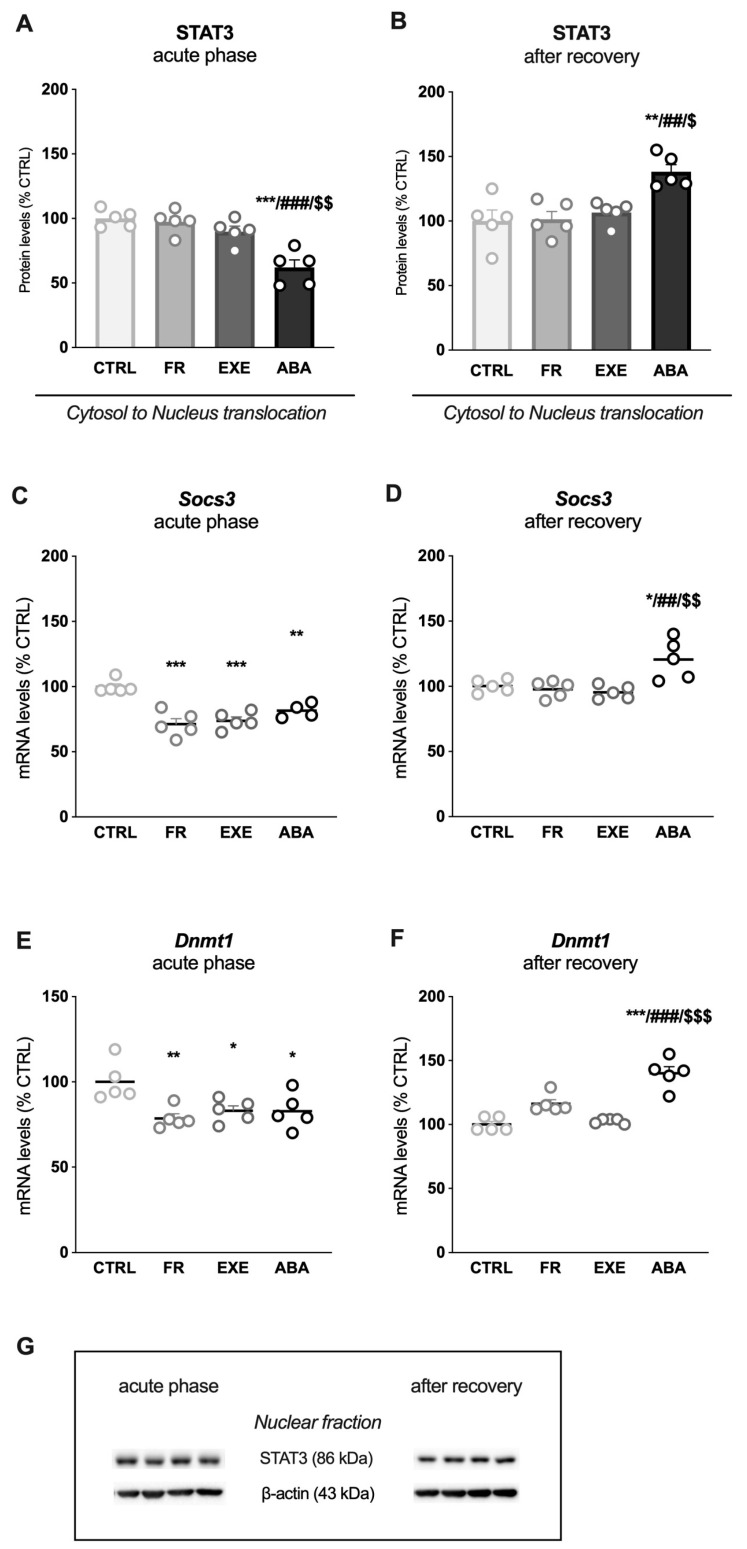
Effects of the ABA paradigm induction on STAT3 cytosol-to-nucleus translocation and on STAT3-dependent genes expression in the ventral hippocampus. The ratio between nuclear and cytosolic STAT3 protein levels is shown in the acute phase of the phenotype (**A**) and after a period of bodyweight recovery (**B**). *Socs3* mRNA levels were evaluated in the acute phase of the disorder (**C**) and after a period of bodyweight recovery (**D**). *Dnmt1* mRNA levels were evaluated in the acute phase of the disorder (**E**) and after a period of bodyweight recovery (**F**). Data are expressed in scatter plot bar graphs as % of CTRL animals and represent the mean ± SEM of five rats per group. Representative immunoblots for each protein are shown in panel (**G**). * *p* < 0.05, ** *p* < 0.01, *** *p* < 0.001 vs. CTRL; ## *p* < 0.01, ### *p* < 0.001 vs. FR; $ *p* < 0.05, $$ *p* < 0.01, $$$ *p* < 0.001 vs. EXE. (Two-way ANOVA followed by Tukey’s multiple comparisons test). CTRL: control; FR: food-restricted; EXE: exercise; ABA: activity-based anorexia.

**Figure 4:**
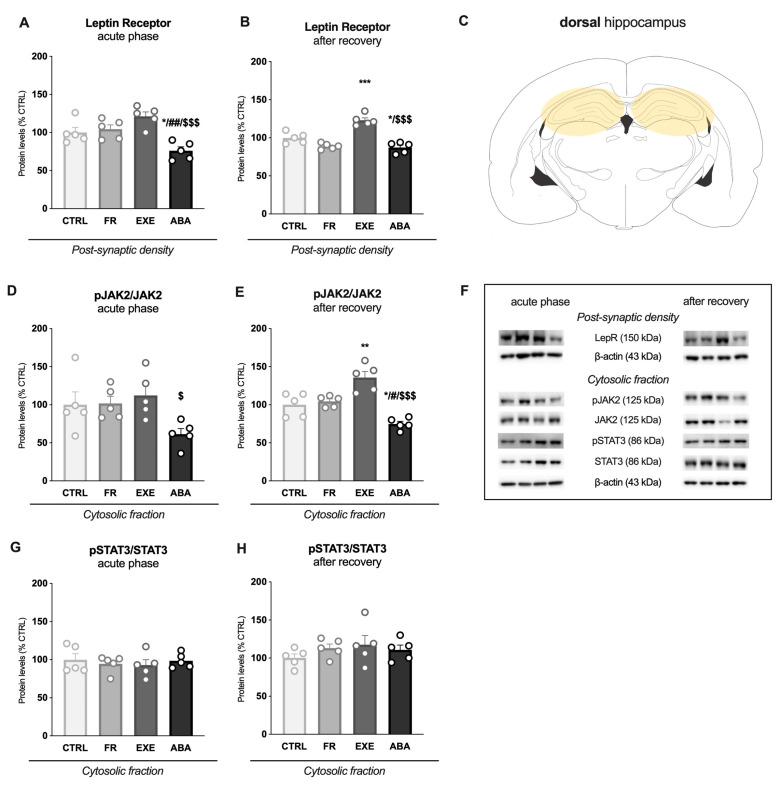
**Effects of the ABA paradigm induction on the leptin receptor and signaling in the dorsal hippocampus.** LepR levels, measured in the PSD, are shown in the acute phase of the pathology (**A**) and after a 7-day recovery period (**B**). Levels of phosphorylation and total protein expression of JAK2 represented as a ratio of phospho(p)-JAK2 in Tyr1007/1008 over total JAK2 and levels of phosphorylation and total protein expression of STAT3 represented as a ratio of phospho(p)-STAT3 in Tyr705 over total STAT3 were measured in the cytosolic fraction in the acute phase of the phenotype (**D**,**G**) and after a period of bodyweight recovery (**E**,**H**). Data are expressed in scatter plot bar graphs as % of CTRL animals and represent the mean ± SEM of five rats per group. Representative immunoblots for each protein are shown in panel (**F**). Panel (**C**) shows the representative image of the subregion of interest (dHip: dorsal hippocampus). * *p* < 0.05, ** *p* < 0.01, *** *p* < 0.001 vs. CTRL; # *p* < 0.05, ## *p* < 0.01 vs. FR; $ *p* < 0.05, $$$ *p* < 0.01 vs. EXE. (Two-way ANOVA followed by Tukey’s multiple comparisons test). CTRL: control; FR: food-restricted; EXE: exercise; ABA: activity-based anorexia.

**Figure 5:**
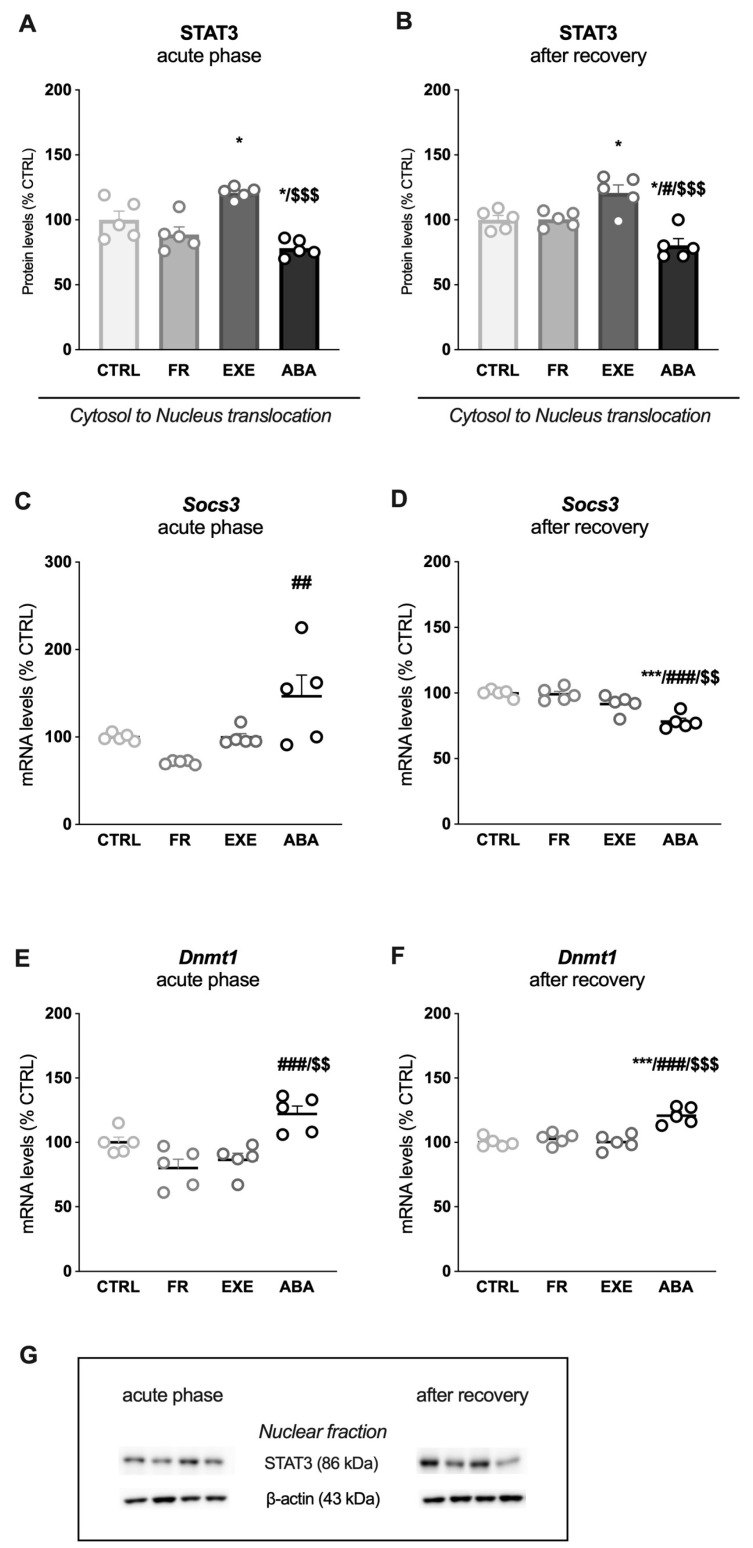
Effects of the ABA paradigm induction on STAT3 cytosol-to-nucleus translocation and on STAT3-dependent genes expression in the dorsal hippocampus. The ratio between nuclear and cytosolic STAT3 protein levels is shown in the acute phase of the phenotype (**A**) and after a period of bodyweight recovery (**B**). *Socs3* mRNA levels were evaluated in the acute phase of the disorder (**C**) and after a period of bodyweight recovery (**D**). *Dnmt1* mRNA levels were evaluated in the acute phase of the disorder (**E**) and after a period of bodyweight recovery (**F**). Data are expressed in scatter plot bar graphs as % of CTRL animals and represent the mean ± SEM of five rats per group. Representative immunoblots for each protein are shown in panel (**G**). * *p* < 0.05, *** *p* < 0.001 vs. CTRL; # *p* < 0.05, ## *p* < 0.01; ### *p* < 0.001 vs. FR; $$ *p* < 0.01, $$$ *p* < 0.001 vs. EXE. (Two-way ANOVA followed by Tukey’s multiple comparisons test). CTRL: control; FR: food-restricted; EXE: exercise; ABA: activity-based anorexia.

Immunocomplexes were detected by chemiluminescence using the Chemidoc MP Imaging System (Bio-Rad Laboratories, Segrate, Italy, RRID: SCR_019037) and analyzed using the Image Lab 6.1 software (Bio-Rad Laboratories). Gels were run two times each, and the final results represent the average from two different runs. A correction factor was used to average different gels: correction factor gel B = average of (OD protein of interest/OD b-actin for each sample loaded in gel A)/(OD protein of interest/OD b-actin for the same sample loaded in gel B) [47]. Full-size original cropped immunoblots related to the protein expression levels evaluated in the present study are enclosed in Supplementary Figures S1–S4 and representative immunoblots for each protein are shown in Figure 2F, Figure 3G, Figure 4F and Figure 5G.

### 2.5. mRNA Isolation and Real-Time PCR Analysis

Total RNA was isolated from vHip and dHip tissues in the TissueLyzer (30 Hz, 30 s) by single-step guanidinium isothiocyanate/phenol extraction via PureZol RNA isolation reagent (Bio-Rad Laboratories, Segrate, Milan, Italy) and quantified by spectrophotometric analysis. Samples were processed for real-time reverse transcription polymerase chain reaction (real-time PCR) to assess mRNA levels as previously reported [48].

RT-PCR analysis was performed to assess Socs3 and Dnmt1 mRNA levels. Data were analyzed with the comparative threshold cycle (DDCt) using 36B4 as the internal standard. Primers and probes for *SOCS3* were purchased from Applied Biosystem, Foster City, California (SOCS3: ID Rn00585674_s1). Primers and probes for *Dnmt1* and *36B4* were purchased from Eurofins MWG-Operon. Their sequences are shown below:

1. *Dnmt1*: forward primer 5′- GCGCTCATTGGCTTTTCTAC-3′, reverse primer 5′- CTCGACCACAATCTTGCTGA-3′, probe 5′- AGCCCAGAGTATGCACCAAT-3′;
2. *36B4*: forward primer 5′-TCAGTGCCTCACTCCATCAT-3′, reverse primer 5′-AGGAAGGCCTTGACCTTTTC-3′, probe 5′-TGGATACAAAAGGGTCCTGG-3′.

### 2.6. Statistical Analysis

Data were collected in individual animals (independent determinations) and are presented as means ± standard errors. Behavioral changes were analyzed by a two-way ANOVA with repeated measures followed by Bonferroni’s multiple comparisons test; for further details, please refer to [39]. Molecular changes were analyzed by a two-way ANOVA, testing physical activity (sedentary vs. exercise) and food availability (food *ad libitum* vs. food restriction) as independent variables at the two different time points analyzed, PND42 or PND49. When dictated by relevant interaction terms, Tukey’s multiple comparisons test was applied to identify differences among individual groups of rats. Detailed statistics, such as F and p values of independent variables of the two-way ANOVA, are reported in Supplementary Tables S3–S6. 

Pearson’s product-moment coefficients (r) and linear regression analyses (R^2^) were determined to study potential correlations between behavioral outcomes induced by the ABA procedure and molecular changes. Subjects were eliminated from the final dataset if their data deviated from the mean by 2 SDs. Prism 9.5.0 (GraphPad Software, Prism v9.5.0, San Diego, CA, USA) was used to analyze all the data. Significance for all tests was assumed at *p* < 0.05. 

## 3. Results

As previously published, the combination of food restriction and running activity causes the development of the AN phenotype in the ABA animals by reaching the maximum-allowed weight loss combined with the hyperactive behavior at PND42, representative of the acute phase [39]. In particular, at this time point, ABA rats showed reduced bodyweight versus CTRL (−65%, *p* < 0.001) and EXE (−54%, *p* < 0.001) rats; interestingly, even though they ate the same amount of food (FR: 9 g; ABA: 8 g), ABA rats displayed reduced bodyweight even compared to FR rats (−22%, *p* < 0.01). This difference is due to the hyperactive behavior displayed by ABA rats on the wheels: starting from the beginning of the food restriction paradigm, ABA rats increased their running activity over days and at PND 42, they ran a significantly increased total distance compared to EXE rats (+16,982 m vs. EXE, *p* < 0.01). 

### 3.1. Effects of the Anorexic Phenotype Induction on Circulating Leptin Levels

Leptin plasma levels were reduced in FR, EXE and ABA animals compared to CTRL rats (Figure 1B left) in the acute phase of the disorder. Moreover, when compared to EXE animals, ABA rats exhibited further diminished leptin plasma levels. On the other hand, after bodyweight recovery, FR and EXE leptin plasma levels were restored back to CTRL levels (Figure 1B right), whereas ABA animals showed a significant increase in leptin plasma levels that exceeded that of CTRL rats. Interestingly, as shown in Figure 1C,D, Pearson’s correlation analysis showed a positive correlation between leptin plasma levels and food intake and bodyweight in FR and ABA animals, while there was a negative correlation between leptin plasma levels and food intake in EXE animals.

### 3.2. Activity-Based Anorexia Alters Intracellular Leptin Receptor Signaling in the Ventral Hippocampus

To investigate whether ABA induction modulates the LepR signaling in the vHip, we measured the LepR/JAK2/STAT3 expression and phosphorylation levels among the different experimental groups at both time points. As illustrated in Figure 2 panel A, in the acute phase, ABA rats showed a reduction in LepR protein levels whereas, following bodyweight recovery (Figure 2B), the ABA group exhibited a significant increase in its expression exceeding PND49 control levels. No changes were observed in FR and EXE rats at both time points (Figure 2A,B). 

Then, to provide insights on downstream kinase activation, we measured the ratio between the phosphorylated and total expression levels of JAK2 (pJAK2_(Tyr1007/1008)_/JAK2) in the cytosolic fraction at both time points. At PND42, the combination of food restriction and exercise significantly reduced the ratio compared to the CTRL, FR and EXE groups; while exercise per sé showed an increase of pJAK2_(Tyr1007/1008)_/JAK2 compared to the CTRL group (Figure 2D). At PND49, the ABA group exhibited an increase in pJAK2_(Tyr1007/1008)_/JAK2 ratio compared to all other groups (Figure 2E), while no changes were observed in the FR and EXE animals. Since JAK2 activates STAT3 to regulate energy homeostasis [8] and synaptic plasticity in the hippocampus [49,50], we analyzed the activation levels of STAT3, measured as the ratio between the phosphorylated and total expression levels of STAT3 (pSTAT3_(Tyr705)_/STAT3), in the cytosolic fraction. At PND42, STAT3 activation was reduced in the FR, EXE and ABA groups (Figure 2G). On the contrary, at PND49, only the combination of food restriction and physical activity increased the pSTAT3_(Tyr705)_/STAT3 ratio (Figure 2H). Since STAT3 exerts its transcriptional activity upon translocating into the nucleus [51], we evaluated the ratio of STAT3 expression levels in the cytosolic versus nuclear fraction as an index of STAT3 translocation from the cytosol to the nucleus. At PND42, only the combination of food restriction and exercise significantly reduced STAT3 translocation (Figure 3A). Conversely, at PND49, ABA rats displayed a significant increase in the nucleus/cytosol ratio of STAT3, whereas no changes were observed in FR and EXE rats (Figure 3B).

To have a readout of the translational activity of STAT3, we evaluated the impact of the anorexic phenotype on the expression of two STAT3-dependent genes: *Socs3*, a protein that terminates JAK-STAT cytokine signaling pathway activity [52] and *Dnmt1*, a DNA methyl transferase that controls synaptic plasticity [53]. In our experimental condition, *Socs3* and *Dnmt1* gene expression were reduced in the FR, EXE and ABA groups at PND42 (Figure 3C and Figure 3E, respectively). Conversely, after weight recovery, *Socs3* and *Dnmt1* mRNA levels were increased only in the ABA group, in line with STAT3 translocation (Figure 3D and Figure 3F, respectively).

### 3.3. Activity-Based Anorexia Alters Intracellular Leptin Receptor Signaling in the Dorsal Hippocampus

Since ventral and dorsal subregions of the hippocampus play different roles, we evaluated the LepR/JAK2/STAT3 pathway also in the dHip.

At PND42, the ABA rats showed reduced LepR protein levels in the post-synaptic density (Figure 4A), which persisted at least until PND49. Interestingly, exercise per sé increased LepR levels at PND49, a week after the last day of exercise (Figure 4B). Next, we analyzed the activation levels of JAK2 and STAT3 in the cytosolic fraction of dHip. At PND42, we observed a reduced activation of the pJAK2_(Tyr1007/1008)_/JAK2 ratio in the ABA group (Figure 4D) while, at PND49, the reduction of JAK2 activation in the ABA group was significant compared to all the other experimental groups (Figure 4E). Moreover, the EXE group showed increased JAK2 activation compared to the CTRL group (Figure 4E). No significant changes were observed in the pSTAT3_(Tyr705)_/STAT3 ratio at both time points (Figure 4G,H).

Then, we evaluated STAT3 expression in the cytosolic fraction versus nuclear expression as an index of translocation. STAT3 translocation was reduced in the ABA group while it increased in the EXE group at both time points (Figure 5A,B). The analysis of STAT3-dependent genes revealed a significant increase in *Socs3* mRNA levels only in ABA animals measured in the acute phase (Figure 5C), whereas, after bodyweight recovery, its levels were reduced only in ABA rats (Figure 5D). Moreover, at PND42, *Dnmt1* mRNA levels were increased only in the ABA group, an effect that persisted after bodyweight recovery (Figure 5E,F).

## 4. Discussion

In our experiments, we observed a biphasic trend in blood leptin levels, with a marked reduction at PND 42 and an increase that exceeded the CTRL levels after one week. We also showed long-lasting changes in the LepR/JAK2/STAT3 signaling pathway in the dHip and vHip of ABA rats which, similarly, reduced immediately after the establishment of the anorexic phenotype but opposite in the two subregions one week later. We believe that such alterations might contribute to driving the transition from adaptation to starvation toward maladaptive alterations that are still observed even when bodyweight is recovered. Our data could help explain the hippocampal-dependent structural and functional impairments observed in the ABA model (Mottarlini et al., unpublished data) [54] and in patients with AN [55,56,57].

Among the different circulating signals reaching the brain from the periphery and that regulate food consumption and energy balance as fundamental mechanisms to maintain physiological bodyweight [35,40], leptin plays a crucial role in AN as evidenced by its reduced levels in underweight patients [33,58]. In clinical settings, leptin has been proposed as an indicator of the nutritional status since it provides an accurate measure of the adipose tissue storages [59] and it predicts AN independently from the body mass index [60]. Refeeding and weight recovery in patients with AN determine a transition from low to normal circulating leptin levels; however, the extent of this hormonal recovery is different as it depends on the age and timing of sampling. Interestingly, leptin levels not completely restored at discharge favor weight loss in the short-term remission [61]; however, higher than physiological levels of leptin predict further weight loss after 1 year [19,20]. In line with clinical data and evidence in rodents [62,63], in our cohort of adolescent female rats, we showed that leptin plasma levels were greatly reduced at PND 42. Hypoleptinemia seems to be due to food restriction per sé, further designating leptin as a critical marker of starvation. The effect of physical activity in reducing leptin levels in EXE rats is due to its role in stabilizing appetite in a condition of exercise-induced negative energy imbalance, thus suggesting that its reduction, despite of a lesser extent than that observed in ABA rats, might be associated with the increased metabolic demands imposed by exercise to regulate energy homeostasis [64]. We observed that EXE and FR rats restored the amount of circulating leptin back to CTRL levels following the 7-day recovery period, suggesting its ability to physiologically respond to food consumption and to the normalization of bodyweight. Of note, ABA rats showed a marked increase in leptin levels, even exceeding CTRL levels with regaining weight, suggesting an impaired leptin system functionality. Interestingly, changes in leptin levels in FR and ABA rats positively correlated with food intake and bodyweight loss, sustaining the hypothesis that fluctuations in its levels might contribute to triggering the mechanisms underlying adaptations to starvation. The hyperleptinemia previously demonstrated in clinical evaluations from the Hebebrand’s group during refeeding [19,20], here also observed in an experimental model of AN following recovery of bodyweight, may contribute to explain the patients’ difficulty reaching or maintaining the target weight. In fact, a sudden increase in leptin blood levels may reduce food consumption and an increase in energy expenditure [65,66], thus mediating elevated caloric demands that, in turn, render AN recovered patients more vulnerable to relapse [67].

Among the extra-hypothalamic regions in which leptin exerts its function, we focused our attention on the hippocampus since sizeable reductions in hippocampal subfield volumes have been observed in young, acutely underweight patients with AN and, even more interestingly, a larger reduction of these hippocampal subfields has been predicted by hypoleptinemia in the acute phase of the disorder [55]. We investigated the two main subregions of the Hip, dorsal and ventral, since they play different roles in regulating emotion, stress-related responses and cognitive processes. In particular, the vHip governs the overall affective state and moderates the effects of emotional experience and stress. The dHip, instead, is mainly involved in controlling cognitive functions, including memory, learning and spatial contextual detection [68,69,70]. In addition, both subregions are involved in delaying meal initiation and influencing the amount of food consumed [71,72]. In the vHip, we found that, following the induction of the anorexic phenotype, the refeeding induced a shift from hypo- to over-activation of the membrane expression of the LepR, of JAK2 and STAT3, in the nuclear translocation of STAT3 and in the transcription of STAT3-dependent genes, *Socs3* and *Dnmt1*. Interestingly, since it has been shown that targeted deletion of LepR in the adult Hip of mice is sufficient to induce depressive-like behaviors and alter the coping ability to stressful events [73], we hypothesize that the reduced activation of the LepR/JAK2/STAT3 pathway in ABA rats in the acute phase of the disorder might contribute to explain the increased anxiety and depressive-like phenotype observed in rats exposed to the combination of reduced energy intake and intense wheel activity [74,75]. From a clinical standpoint, this is of interest since patients with AN suffer from a significant number of psychiatric comorbidities, such as anxiety- and depressive-like symptoms [73,76,77,78], which are rapidly improved by metreleptin treatment [29,30]. The reduced levels of *Socs3*, the inhibitor of JAK2/STAT3, and of *Dnmt1*, crucial for the maintenance of DNA methylation [79], in ABA rats sacrificed in the acute phase might reflect an adaptive response to restore the physiological activity of the pathway via reducing the negative feedback and the methylation-induced repression of transcription. On the contrary, the increased expression of *Socs3* and *Dnmt1* observed following recovery might be a mechanism set in motion by the cells to counteract the hyperactivation of the pathway.

In the dHip, the ABA condition reduced the membrane expression of the LepR, the activation of JAK2 and the nuclear translocation of STAT3, effects that persisted even following the 7-day recovery period. Since the regulatory action of leptin in the Hip is pro-cognitive, acting on the strength and functionality of synaptic connections [49], a reduced LepR/JAK2/STAT3 pathway in ABA rats might contribute, in turn, to explaining the altered homeostasis of the glutamate synapse, the reduced spine density and the spatial memory impairment that we have previously observed in ABA rats in the spatial order object recognition test performed at both PND42 and PND49 (Mottarlini et al., unpublished data). Moreover, the increased levels of *Socs3* and *Dnmt1* in the acute phase, an effect that might be induced by signals other than leptin itself, such as neuroinflammatory regulators [80,81], might contribute to further reducing the LepR pathway activity. Of note, despite weight recovery, the persistent reduction in the LepR/JAK2/STAT3 pathway in ABA rats reduced *Socs3* expression probably via Dnmt1-induced hypermethylation of *Socs3* promoter [82], thus reflecting an adaptive response to restoring the physiological activity of the pathway. This biphasic expression of *Socs3* in the dHip adds complexity to the molecular dysregulation induced by the anorexic phenotype on the leptin signaling that may contribute to inaccurate information processing and, ultimately, affect hippocampal-dependent memory and associative learning observed in ABA rats (Mottarlini et al., unpublished data) and in patients with AN [83,84].

Since the maturation of both dHip and vHip is protracted during late adolescence [85], our data corroborate the hypothesis that the ABA paradigm interferes with the hippocampal maturational trajectory via altered periphery-to-brain crosstalk that might ultimately modulate cognitive function, emotional behavior and associative learning, thus contributing to altering stimuli related to food salience [54,86,87,88]. The changes in the LepR/JAK2/STAT3 pathway, which persist even after bodyweight restoration, strongly suggest that the leptin signaling pathway is involved in the mechanisms set in motion by hyperactivity-induced starvation and might drive the transition from adaptation to starvation toward persistent maladaptive alterations. Despite this evidence, we cannot rule out the possibility that other peripherally-produced factors, such as myokines, adipokines and hormonal responses [23,89,90], might contribute to the molecular alterations induced by the combination of low energy intake and hyperactivity in the dHip and vHip. Moreover, the precise underlying mechanisms of this transition, which we hypothesized took place also by recruiting other neurotransmitter systems [(i.e., the glutamatergic system, Mottarlini et al., unpublished data, [91,92]], still need to be completely understood. Even though in the acute phase of the disorder the single conditions of FR and EXE modulate leptin plasma levels, this does not happen in the brain where, at least in the vHip and dHip, we observed an altered activation of the LepR pathway only in ABA rats. Moreover, a previous report indicates that FR and EXE alone induced changes in dendritic arbors in the dHip and vHip upon age-matched cohorts of rats [86]: our data suggest that the LepR/JAK2/STAT3 pathway may not be involved in such regulatory mechanisms.

We are aware that this paper holds some limitations. Since LepR is expressed also in astrocytes [93] and its deletion in GFAP-positive astrocytes reduces basal synaptic transmission [94], we cannot infer the herein-shown changes in the LepR pathway to be mainly neuronal or glial. Moreover, our data are limited to dHip and vHip whereas the possibility exists that the LepR/JAK2/STAT3 signaling pathway might be dysregulated in other extra-hypothalamic brain regions, such as the prefrontal cortex, ventral tegmental area and nucleus accumbens.

## 5. Conclusions

Leptin plays a significant role in the clinical symptomatology and the progression of AN; indeed, it is also considered a risk factor for relapse episodes [14,95] and four variants in the sequence of the coding region of the leptin gene have been identified in acutely ill AN patients [96]. Our data add evidence to the hypothesis that hypoleptinemia could be an initial driver of the adaptations set in motion by the body in a condition of starvation. In parallel, reduced activation of the brain LepR pathway in the acute phase of AN in both hippocampal subregions may account for altered cognitive and emotional processes, typical of patients with AN. On the other hand, following the recovery period, persistent low activation in the dHip might fuel the hypoleptinemia-induced alteration of aberrant salience driving maladaptive learning, while the concomitant increased activation of the pathway in the vHip might contribute to explaining the improvement of anxiety-like behavior previously observed in recovered ABA rats [54]. Thus, the herein-shown fluctuations of circulating leptin and its signaling in ABA rats might contribute to set the stage for the transition to AN via persistent, albeit different, changes in the dHip and vHip LepR/JAK2/STAT3 signaling pathway that cannot be ascribed to a generalized effect of malnutrition or exercise alone. Therefore, future studies on the maladaptive relationship among peripheral metabolic changes and brain functions are needed in human cohorts of AN patients to provide further insights and develop targeted and symptom-specific treatments [97].

## Data Availability

Dataset available on request from the corresponding author. The data are not publicly available due to privacy restriction.

## Supplementary Materials

The following supporting information can be downloaded at: https://www.mdpi.com/article/10.3390/nu16081171/s1, Figures S1–S4: Example of full-size cropped immunoblots; Tables S1, S4–S7: Details of statistical analysis; Tables S2 and S3: Details of statistical analysis.

## References

[1] Barrios-Correa A.A., Estrada J.A., Contreras I. (2018). Leptin Signaling in the Control of Metabolism and Appetite: Lessons from Animal Models. J. Mol. Neurosci..

[2] Chan J.L., Blüher S., Yiannakouris N., Suchard M.A., Kratzsch J., Mantzoros C.S. (2002). Regulation of Circulating Soluble Leptin Receptor Levels By Gender, Adiposity, Sex Steroids, and Leptin. Diabetes.

[3] Liu Z., Xiao T., Liu H. (2023). Leptin Signaling and Its Central Role in Energy Homeostasis. Front. Neurosci..

[4] Obradovic M., Sudar-Milovanovic E., Soskic S., Essack M., Arya S., Stewart A.J., Gojobori T., Isenovic E.R. (2021). Leptin and Obesity: Role and Clinical Implication. Front. Endocrinol..

[5] Liu H., Guo X., Jiang K., Shi B., Liu L., Hou R., Chen G., Farag M.A., Yan N., Liu L. (2024). Dietary Polyphenols Regulate Appetite Mechanism via Gut-Brain Axis and Gut Homeostasis. Food Chem..

[6] Bjørbaek C., Uotani S., da Silva B., Flier J.S. (1997). Divergent Signaling Capacities of the Long and Short Isoforms of the Leptin Receptor. J. Biol. Chem..

[7] Banks A.S., Davis S.M., Bates S.H., Myers M.G. (2000). Activation of Downstream Signals by the Long Form of the Leptin Receptor. J. Biol. Chem..

[8] Bates S.H., Stearns W.H., Dundon T.A., Schubert M., Tso A.W.K., Wang Y., Banks A.S., Lavery H.J., Haq A.K., Maratos-Flier E. (2003). STAT3 Signalling Is Required for Leptin Regulation of Energy Balance but Not Reproduction. Nature.

[9] Patterson C.M., Leshan R.L., Jones J.C., Myers M.G. (2011). Molecular Mapping of Mouse Brain Regions Innervated by Leptin Receptor-Expressing Cells. Brain Res..

[10] Harvey J. (2007). Leptin Regulation of Neuronal Excitability and Cognitive Function. Curr. Opin. Pharmacol..

[11] Farr S.A., Banks W.A., Morley J.E. (2006). Effects of Leptin on Memory Processing. Peptides.

[12] Guo M., Lu Y., Garza J.C., Li Y., Chua S.C., Zhang W., Lu B., Lu X.-Y. (2012). Forebrain Glutamatergic Neurons Mediate Leptin Action on Depression-like Behaviors and Synaptic Depression. Transl. Psychiatry.

[13] Lawson E.A., Miller K.K., Blum J.I., Meenaghan E., Misra M., Eddy K.T., Herzog D.B., Klibanski A. (2012). Leptin Levels Are Associated With Decreased Depressive Symptoms in Women Across the Weight Spectrum, Independent of Body Fat. Clin. Endocrinol..

[14] Hebebrand J., Hildebrandt T., Schlögl H., Seitz J., Denecke S., Vieira D., Gradl-Dietsch G., Peters T., Antel J., Lau D. (2022). The Role of Hypoleptinemia in the Psychological and Behavioral Adaptation to Starvation: Implications for Anorexia Nervosa. Neurosci. Biobehav. Rev..

[15] Scharf I. (2016). The Multifaceted Effects of Starvation on Arthropod Behaviour. Anim. Behav..

[16] van Eeden A.E., van Hoeken D., Hoek H.W. (2021). Incidence, Prevalence and Mortality of Anorexia Nervosa and Bulimia Nervosa. Curr. Opin. Psychiatry.

[17] American Psychiatric Association (2013). DSM-V Diagnostic and Statistical Manual of Mental Disorders.

[18] Zipfel S., Giel K.E., Bulik C.M., Hay P., Schmidt U. (2015). Anorexia Nervosa: Aetiology, Assessment, and Treatment. Lancet Psychiatry.

[19] Hebebrand J., Blum W.F., Barth N., Coners H., Englaro P., Juul A., Ziegler A., Warnke A., Rascher W., Remschmidt H. (1997). Leptin Levels in Patients with Anorexia Nervosa Are Reduced in the Acute Stage and Elevated upon Short-Term Weight Restoration. Mol. Psychiatry.

[20] Holtkamp K., Hebebrand J., Mika C., Grzella I., Heer M., Heussen N., Herpertz-Dahlmann B. (2003). The Effect of Therapeutically Induced Weight Gain on Plasma Leptin Levels in Patients with Anorexia Nervosa. J. Psychiatr. Res..

[21] Mantzoros C., Flier J.S., Lesem M.D., Brewerton T.D., Jimerson D.C. (1997). Cerebrospinal Fluid Leptin in Anorexia Nervosa: Correlation with Nutritional Status and Potential Role in Resistance to Weight Gain1. J. Clin. Endocrinol. Metab..

[22] Monteleone P., Di Lieto A., Tortorella A., Longobardi N., Maj M. (2000). Circulating Leptin in Patients with Anorexia Nervosa, Bulimia Nervosa or Binge-Eating Disorder: Relationship to Body Weight, Eating Patterns, Psychopathology and Endocrine Changes. Psychiatry Res..

[23] Monteleone P., Maj M. (2013). Dysfunctions of Leptin, Ghrelin, BDNF and Endocannabinoids in Eating Disorders: Beyond the Homeostatic Control of Food Intake. Psychoneuroendocrinology.

[24] Hebebrand J., Bulik C.M. (2011). Critical Appraisal of the Provisional DSM-5 Criteria for Anorexia Nervosa and an Alternative Proposal. Int. J. Eat. Disord..

[25] Lob S., Pickel J., Bidlingmaier M., Schaaf L., Backmund H., Gerlinghoff M., Stalla G. (2003). Serum Leptin Monitoring in Anorectic Patients During Refeeding Therapy. Exp. Clin. Endocrinol. Diabetes.

[26] Davis J.F., Choi D.L., Benoit S.C. (2010). Insulin, Leptin and Reward. Trends Endocrinol. Metab..

[27] Irving A.J., Harvey J. (2014). Leptin Regulation of Hippocampal Synaptic Function in Health and Disease. Philos. Trans. R. Soc. Lond. B Biol. Sci..

[28] Holtkamp K., Herpertz-Dahlmann B., Hebebrand K., Mika C., Kratzsch J., Hebebrand J. (2006). Physical Activity and Restlessness Correlate with Leptin Levels in Patients with Adolescent Anorexia Nervosa. Biol. Psychiatry.

[29] Hebebrand J., Milos G., Wabitsch M., Teufel M., Führer D., Bühlmeier J., Libuda L., Ludwig C., Antel J. (2019). Clinical Trials Required to Assess Potential Benefits and Side Effects of Treatment of Patients With Anorexia Nervosa With Recombinant Human Leptin. Front. Psychol..

[30] Milos G., Antel J., Kaufmann L.-K., Barth N., Koller A., Tan S., Wiesing U., Hinney A., Libuda L., Wabitsch M. (2020). Short-Term Metreleptin Treatment of Patients with Anorexia Nervosa: Rapid on-Set of Beneficial Cognitive, Emotional, and Behavioral Effects. Transl. Psychiatry.

[31] Hebebrand J., Hinney A., Antel J. (2023). Could Leptin Substitution Therapy Potentially Terminate Entrapment in Anorexia Nervosa?. Nat. Rev. Endocrinol..

[32] Exner C., Hebebrand J., Remschmidt H., Wewetzer C., Ziegler A., Herpertz S., Schweiger U., Blum W.F., Preibisch G., Heldmaier G. (2000). Leptin Suppresses Semi-Starvation Induced Hyperactivity in Rats: Implications for Anorexia Nervosa. Mol. Psychiatry.

[33] Hillebrand J.J.G., Koeners M.P., de Rijke C.E., Kas M.J.H., Adan R.A.H. (2005). Leptin Treatment in Activity-Based Anorexia. Biol. Psychiatry.

[34] Verhagen L.A.W., Luijendijk M.C.M., Adan R.A.H. (2011). Leptin Reduces Hyperactivity in an Animal Model for Anorexia Nervosa via the Ventral Tegmental Area. Eur. Neuropsychopharmacol..

[35] Foldi C.J. (2023). Taking Better Advantage of the Activity-Based Anorexia Model. Trends Mol. Med..

[36] Park H.-K., Ahima R.S. (2014). Leptin Signaling. F1000Prime Rep..

[37] Day J.J., Sweatt J.D. (2010). DNA Methylation and Memory Formation. Nat. Neurosci..

[38] Egger G., Liang G., Aparicio A., Jones P.A. (2004). Epigenetics in Human Disease and Prospects for Epigenetic Therapy. Nature.

[39] Mottarlini F., Rizzi B., Targa G., Fumagalli F., Caffino L. (2022). Long-Lasting BDNF Signaling Alterations in the Amygdala of Adolescent Female Rats Exposed to the Activity-Based Anorexia Model. Front. Behav. Neurosci..

[40] Gutierrez E. (2013). A Rat in the Labyrinth of Anorexia Nervosa: Contributions of the Activity-Based Anorexia Rodent Model to the Understanding of Anorexia Nervosa. Int. J. Eat. Disord..

[41] Chapman R.H., Stern J.M. (1978). Maternal Stress and Pituitary-Adrenal Manipulations during Pregnancy in Rats: Effects on Morphology and Sexual Behavior of Male Offspring. J. Comp. Physiol. Psychol..

[42] Boakes R.A., Dwyer D.M. (1997). Weight Loss in Rats Produced by Running: Effects of Prior Experience and Individual Housing. Q. J. Exp. Psychol. B.

[43] Tezenas Du Montcel C., Cao J., Mattioni J., Hamelin H., Lebrun N., Ramoz N., Gorwood P., Tolle V., Viltart O. (2023). Chronic Food Restriction in Mice and Increased Systemic Ghrelin Induce Preference for Running Wheel Activity. Psychoneuroendocrinology.

[44] Carrera O., Fraga Á., Pellón R., Gutiérrez E. (2014). Rodent Model of Activity-Based Anorexia. Curr. Protoc. Neurosci..

[45] Paxinos G., Watson C. (2013). The Rat Brain in Stereotaxic Coordinates.

[46] Targa G., Mottarlini F., Rizzi B., Leo D., Caffino L., Fumagalli F. (2023). Dysregulation of AMPA Receptor Trafficking and Intracellular Vesicular Sorting in the Prefrontal Cortex of Dopamine Transporter Knock-Out Rats. Biomolecules.

[47] Caffino L., Verheij M.M.M., Roversi K., Targa G., Mottarlini F., Popik P., Nikiforuk A., Golebiowska J., Fumagalli F., Homberg J.R. (2020). Hypersensitivity to Amphetamine’s Psychomotor and Reinforcing Effects in Serotonin Transporter Knockout Rats: Glutamate in the Nucleus Accumbens. Br. J. Pharmacol..

[48] Caffino L., Mottarlini F., Mingardi J., Zita G., Barbon A., Fumagalli F. (2020). Anhedonic-like Behavior and BDNF Dysregulation Following a Single Injection of Cocaine during Adolescence. Neuropharmacology.

[49] Harvey J. (2022). Food for Thought: Leptin and Hippocampal Synaptic Function. Front. Pharmacol..

[50] Harvey J., Solovyova N., Irving A. (2006). Leptin and Its Role in Hippocampal Synaptic Plasticity. Prog. Lipid Res..

[51] Hübschle T., Thom E., Watson A., Roth J., Klaus S., Meyerhof W. (2001). Leptin-Induced Nuclear Translocation of STAT3 Immunoreactivity in Hypothalamic Nuclei Involved in Body Weight Regulation. J. Neurosci..

[52] Endo T.A., Masuhara M., Yokouchi M., Suzuki R., Sakamoto H., Mitsui K., Matsumoto A., Tanimura S., Ohtsubo M., Misawa H. (1997). A New Protein Containing an SH2 Domain That Inhibits JAK Kinases. Nature.

[53] Halder R., Hennion M., Vidal R.O., Shomroni O., Rahman R.-U., Rajput A., Centeno T.P., van Bebber F., Capece V., Garcia Vizcaino J.C. (2016). DNA Methylation Changes in Plasticity Genes Accompany the Formation and Maintenance of Memory. Nat. Neurosci..

[54] Chowdhury T.G., Fenton A.A., Aoki C. (2021). Effects of Adolescent Experience of Food Restriction and Exercise on Spatial Learning and Open Field Exploration of Female Rats. Hippocampus.

[55] Bahnsen K., Wronski M.-L., Keeler J.L., King J.A., Preusker Q., Kolb T., Weidner K., Roessner V., Bernardoni F., Ehrlich S. (2023). Differential Longitudinal Changes of Hippocampal Subfields in Patients with Anorexia Nervosa. Psychiatry Clin. Neurosci..

[56] Collantoni E., Tenconi E., Solmi M., Meneguzzo P., Marzola E., D’Agata F., Gotti S., Daga G.A., Manara R., Favaro A. (2021). Hippocampal Volumes in Anorexia Nervosa at Different Stages of the Disorder. Eur. Eat. Disord. Rev..

[57] Keeler J., Patsalos O., Thuret S., Ehrlich S., Tchanturia K., Himmerich H., Treasure J. (2020). Hippocampal Volume, Function, and Related Molecular Activity in Anorexia Nervosa: A Scoping Review. Expert. Rev. Clin. Pharmacol..

[58] Alzaid H., Simon J.J., Brugnara G., Vollmuth P., Bendszus M., Friederich H.-C. (2024). Hypothalamic Subregion Alterations in Anorexia Nervosa and Obesity: Association with Appetite-Regulating Hormone Levels. Int. J. Eat. Disord..

[59] Arner E., Westermark P.O., Spalding K.L., Britton T., Rydén M., Frisén J., Bernard S., Arner P. (2010). Adipocyte Turnover: Relevance to Human Adipose Tissue Morphology. Diabetes.

[60] Föcker M., Timmesfeld N., Scherag S., Bühren K., Langkamp M., Dempfle A., Sheridan E.M., de Zwaan M., Fleischhaker C., Herzog W. (2011). Screening for Anorexia Nervosa via Measurement of Serum Leptin Levels. J. Neural Transm..

[61] Dardennes R., Tolle V., Lavoisy G., Grouselle D., Alanbar N., Duriez P., Gorwood P., Ramoz N., Epelbaum J. (2021). Lower Leptin Level at Discharge in Acute Anorexia Nervosa Is Associated with Early Weight-Loss. Eur. Eat. Disord. Rev..

[62] Scherma M., Satta V., Collu R., Boi M.F., Usai P., Fratta W., Fadda P. (2017). Cannabinoid CB1 /CB2 Receptor Agonists Attenuate Hyperactivity and Body Weight Loss in a Rat Model of Activity-Based Anorexia. Br. J. Pharmacol..

[63] Gilman T.L., Owens W.A., George C.M., Metzel L., Vitela M., Ferreira L., Bowman M.A., Gould G.G., Toney G.M., Daws L.C. (2019). Age- and Sex-Specific Plasticity in Dopamine Transporter Function Revealed by Food Restriction and Exercise in a Rat Activity-Based Anorexia Paradigm. J. Pharmacol. Exp. Ther..

[64] de Assis G.G., Murawska-Ciałowicz E. (2023). Exercise and Weight Management: The Role of Leptin-A Systematic Review and Update of Clinical Data from 2000-2022. J Clin Med.

[65] Kaye W.H., Gwirtsman H.E., Obarzanek E., George T., Jimerson D.C., Ebert M.H. (1986). Caloric Intake Necessary for Weight Maintenance in Anorexia Nervosa: Nonbulimics Require Greater Caloric Intake than Bulimics. Am. J. Clin. Nutr..

[66] Weltzin T.E., Fernstrom M.H., Hansen D., McConaha C., Kaye W.H. (1991). Abnormal Caloric Requirements for Weight Maintenance in Patients with Anorexia and Bulimia Nervosa. Am. J. Psychiatry.

[67] Holtkamp K., Hebebrand J., Mika C., Heer M., Heussen N., Herpertz-Dahlmann B. (2004). High Serum Leptin Levels Subsequent to Weight Gain Predict Renewed Weight Loss in Patients with Anorexia Nervosa. Psychoneuroendocrinology.

[68] Eijkenboom M., Van Der Staay F.J. (1999). Spatial Learning Deficits in Rats after Injection of Vincristine into the Dorsal Hippocampus. Neuroscience.

[69] Fanselow M.S., Dong H.-W. (2010). Are The Dorsal and Ventral Hippocampus Functionally Distinct Structures?. Neuron.

[70] Meléndez D.M., Nordquist R.E., Vanderschuren L.J.M.J., van der Staay F.-J. (2020). Spatial Memory Deficits after Vincristine-Induced Lesions to the Dorsal Hippocampus. PLoS ONE.

[71] Ragu-Varman D., Macedo-Mendoza M., Labrada-Moncada F.E., Reyes-Ortega P., Morales T., Martínez-Torres A., Reyes-Haro D. (2019). Anorexia Increases Microglial Density and Cytokine Expression in the Hippocampus of Young Female Rats. Behav. Brain Res..

[72] Webster M., Ungerleider L., Bachevalier J. (1991). Connections of Inferior Temporal Areas TE and TEO with Medial Temporal- Lobe Structures in Infant and Adult Monkeys. J. Neurosci..

[73] Guo M., Huang T.-Y., Garza J.C., Chua S.C., Lu X.-Y. (2013). Selective Deletion of Leptin Receptors in Adult Hippocampus Induces Depression-Related Behaviors. Int. J. Neuropsychopharmacol..

[74] Wable G.S., Min J.-Y., Chen Y.-W., Aoki C. (2015). Anxiety Is Correlated with Running in Adolescent Female Mice Undergoing Activity-Based Anorexia. Behav. Neurosci..

[75] Aoki C., Santiago A.N. (2022). Pathway-Specific GABAergic Inhibition Contributes to the Gain of Resilience against Anorexia-like Behavior of Adolescent Female Mice. Front. Behav. Neurosci..

[76] Anckarsäter H., Hofvander B., Billstedt E., Gillberg I.C., Gillberg C., Wentz E., Råstam M. (2012). The Sociocommunicative Deficit Subgroup in Anorexia Nervosa: Autism Spectrum Disorders and Neurocognition in a Community-Based, Longitudinal Study. Psychol. Med..

[77] Bora E., Köse S. (2016). Meta-Analysis of Theory of Mind in Anorexia Nervosa and Bulimia Nervosa: A Specific İmpairment of Cognitive Perspective Taking in Anorexia Nervosa?. Int. J. Eat. Disord..

[78] Kaye W.H., Bulik C.M., Thornton L., Barbarich N., Masters K. (2004). Comorbidity of Anxiety Disorders with Anorexia and Bulimia Nervosa. Am. J. Psychiatry.

[79] Chen Z., Zhang Y. (2020). Role of Mammalian DNA Methyltransferases in Development. Annu. Rev. Biochem..

[80] Abella V., Scotece M., Conde J., Pino J., Gonzalez-Gay M.A., Gómez-Reino J.J., Mera A., Lago F., Gómez R., Gualillo O. (2017). Leptin in the Interplay of Inflammation, Metabolism and Immune System Disorders. Nat. Rev. Rheumatol..

[81] Da Ré C., Souza J.M., Fróes F., Taday J., Dos Santos J.P., Rodrigues L., Sesterheim P., Gonçalves C.A., Leite M.C. (2020). Neuroinflammation Induced by Lipopolysaccharide Leads to Memory Impairment and Alterations in Hippocampal Leptin Signaling. Behav. Brain Res..

[82] Dees C., Pötter S., Zhang Y., Bergmann C., Zhou X., Luber M., Wohlfahrt T., Karouzakis E., Ramming A., Gelse K. (2020). TGF-β-Induced Epigenetic Deregulation of SOCS3 Facilitates STAT3 Signaling to Promote Fibrosis. J. Clin. Invest..

[83] Foerde K., Steinglass J.E. (2017). Decreased Feedback Learning in Anorexia Nervosa Persists after Weight Restoration. Int. J. Eat. Disord..

[84] Guardia D., Carey A., Cottencin O., Thomas P., Luyat M. (2013). Disruption of Spatial Task Performance in Anorexia Nervosa. PLoS ONE.

[85] Chen Y.-W., Akad A., Aderogba R., Chowdhury T.G., Aoki C. (2018). Dendrites of the Dorsal and Ventral Hippocampal CA1 Pyramidal Neurons of Singly Housed Female Rats Exhibit Lamina-Specific Growths and Retractions during Adolescence That Are Responsive to Pair Housing. Synapse.

[86] Aoki C., Chowdhury T.G., Wable G.S., Chen Y.-W. (2017). Synaptic Changes in the Hippocampus of Adolescent Female Rodents Associated with Resilience to Anxiety and Suppression of Food Restriction-Evoked Hyperactivity in an Animal Model for Anorexia Nervosa. Brain Res..

[87] Chowdhury T.G., Barbarich-Marsteller N.C., Chan T.E., Aoki C. (2014). Activity-Based Anorexia Has Differential Effects on Apical Dendritic Branching in Dorsal and Ventral Hippocampal CA1. Brain Struct. Funct..

[88] Chowdhury T.G., Ríos M.B., Chan T.E., Cassataro D.S., Barbarich-Marsteller N.C., Aoki C. (2014). Activity-Based Anorexia during Adolescence Disrupts Normal Development of the CA1 Pyramidal Cells in the Ventral Hippocampus of Female Rats. Hippocampus.

[89] Berner L.A., Brown T.A., Lavender J.M., Lopez E., Wierenga C.E., Kaye W.H. (2019). Neuroendocrinology of Reward in Anorexia Nervosa and Bulimia Nervosa: Beyond Leptin and Ghrelin. Mol. Cell Endocrinol..

[90] Amorim T., Khiyami A., Latif T., Fazeli P.K. (2023). Neuroendocrine Adaptations to Starvation. Psychoneuroendocrinology.

[91] Mottarlini F., Bottan G., Tarenzi B., Colciago A., Fumagalli F., Caffino L. (2020). Activity-Based Anorexia Dynamically Dysregulates the Glutamatergic Synapse in the Nucleus Accumbens of Female Adolescent Rats. Nutrients.

[92] Mottarlini F., Targa G., Bottan G., Tarenzi B., Fumagalli F., Caffino L. (2022). Cortical Reorganization of the Glutamate Synapse in the Activity-Based Anorexia Rat Model: Impact on Cognition. J. Neurochem..

[93] Hsuchou H., Pan W., Barnes M.J., Kastin A.J. (2009). Leptin Receptor mRNA in Rat Brain Astrocytes. Peptides.

[94] Naranjo V., Contreras A., Merino B., Plaza A., Lorenzo M.P., García-Cáceres C., García A., Chowen J.A., Ruiz-Gayo M., Del Olmo N. (2020). Specific Deletion of the Astrocyte Leptin Receptor Induces Changes in Hippocampus Glutamate Metabolism, Synaptic Transmission and Plasticity. Neuroscience.

[95] Hebebrand J., Plieger M., Milos G., Peters T., Hinney A., Antel J. (2024). Does Hypoleptinemia Trigger Entrapment in Anorexia Nervosa? Etiological and Clinical Considerations. Eur. Eat. Disord. Rev..

[96] Rajcsanyi L.S., Zheng Y., Herpertz-Dahlmann B., Seitz J., de Zwaan M., Herzog W., Ehrlich S., Zipfel S., Giel K., Egberts K. (2024). Unexpected Identification of Obesity-Associated Mutations in LEP and MC4R Genes in Patients with Anorexia Nervosa. Sci. Rep..

[97] Keeler J.L., Kan C., Treasure J., Himmerich H. (2023). Novel Treatments for Anorexia Nervosa: Insights from Neuroplasticity Research. Eur. Eat. Disord. Rev..

