# Supplementary material for: Anorexia-Induced Hypoleptinemia Drives Adaptations in the JAK2/STAT3 Pathway in the Ventral and Dorsal Hippocampus of Female Rats
Source: Nutrients. 2024 Apr 15;16(8):1171. doi: 10.3390/nu16081171 (PMC11054075; doi:10.3390/nu16081171)
Supplement: Supplementary file 1 [file nutrients-16-01171-s001.zip › nutrients-2920385-supplementary.pdf]

Supplementary figure S1. A Example of full-size cropped immunoblots related to the expression levels of LepR (150 kDa) and β-actin (43 kDa) measured in the whole post-synaptic density of the ventral Hippocampus of CTRL, FR, EXE and ABA rats in the acute phase of the anorexic phenotype (PND 42); **B** Example of full-size cropped immunoblots related to the expression levels of pJAK2 (125 kDa), JAK2 (125 kDa), pSTAT3 (86 kDa), STAT3 (86 kDa) and β-actin (43 kDa) measured in the cytosolic fraction of the ventral Hippocampus of CTRL, FR, EXE and ABA rats in the acute phase of the anorexic phenotype (PND42); **C** Example of full-size cropped immunoblots related to the expression levels of STAT3 (86 kDa) and β-actin (43 kDa) measured in the nuclear fraction of the ventral Hippocampus of CTRL, FR, EXE and ABA rats in the acute phase of the anorexic phenotype (PND42).
1= controls, CTRL; 2= food restricted, FR; 3= exercise, EXE; 4= activity-based anorexia, ABA.

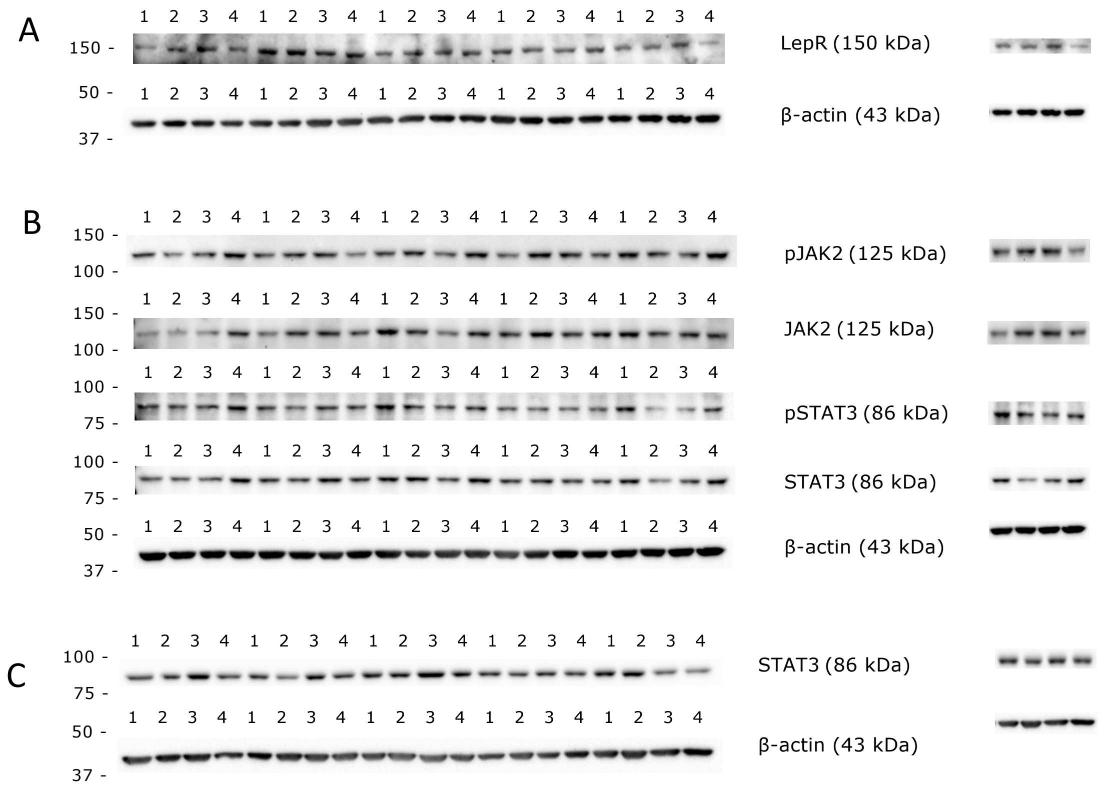

Supplementary figure S2. A Example of full-size cropped immunoblots related to the expression levels of LepR (150 kDa) and β-actin (43 kDa) measured in the whole post-synaptic density of the ventral Hippocampus of CTRL, FR, EXE and ABA rats after the recovery phase of the anorexic phenotype (PND 49); **B** Example of full-size cropped immunoblots related to the expression levels of pJAK2 (125 kDa), JAK2 (125 kDa), pSTAT3 (86 kDa), STAT3 (86 kDa) and β-actin (43 kDa) measured in the cytosolic fraction of the ventral Hippocampus of CTRL, FR, EXE and ABA rats after the recovery phase of the anorexic phenotype (PND 49); **C** Example of full-size cropped immunoblots related to the expression levels of STAT3 (86 kDa) and β-actin (43 kDa) measured in the nuclear fraction of the ventral Hippocampus of CTRL, FR, EXE and ABA rats after the recovery phase of the anorexic phenotype (PND 49).
1= controls, CTRL; 2= food restricted, FR; 3= exercise, EXE; 4= activity-based anorexia, ABA.

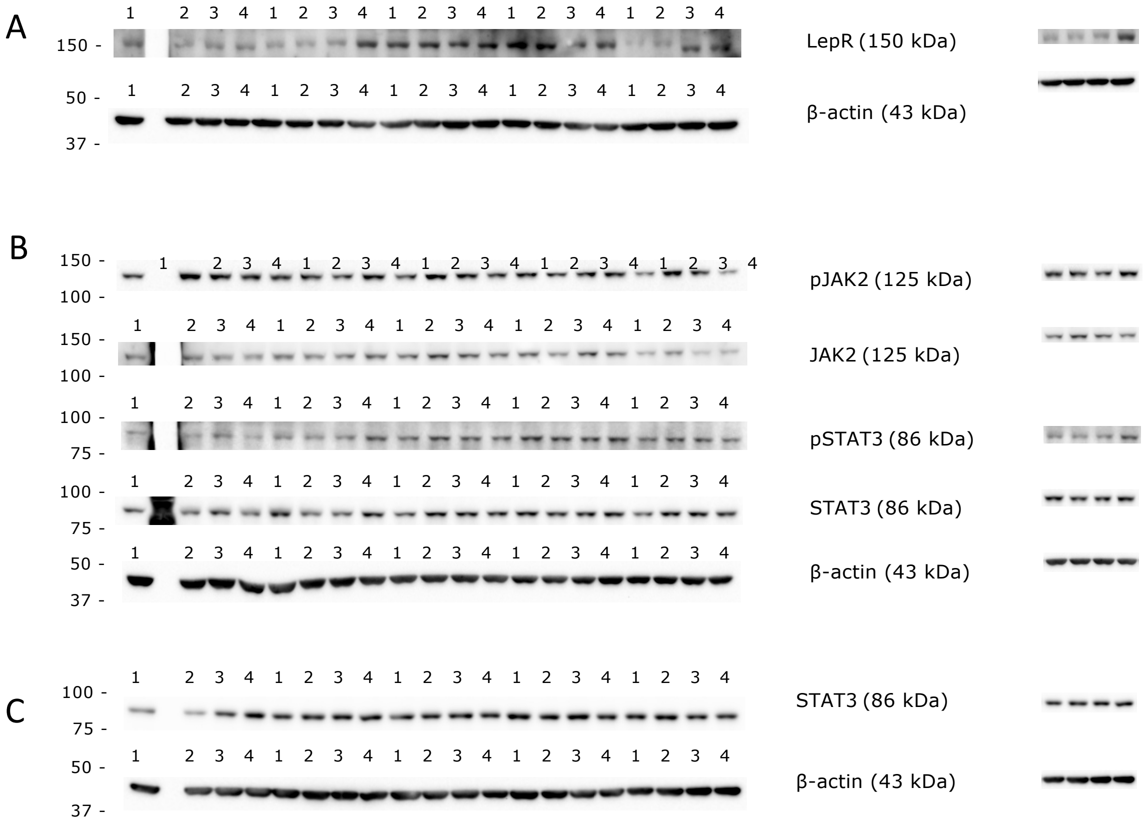

Supplementary figure S3. A Example of full-size cropped immunoblots related to the expression levels of LepR (150 kDa) and β-actin (43 kDa) measured in the whole post-synaptic density of the dorsal Hippocampus of CTRL, FR, EXE and ABA rats in the acute phase of the anorexic phenotype (PND 42); **B** Example of full-size cropped immunoblots related to the expression levels of pJAK2 (125 kDa), JAK2 (125 kDa), pSTAT3 (86 kDa), STAT3 (86 kDa) and β-actin (43 kDa) measured in the cytosolic fraction of the dorsal Hippocampus of CTRL, FR, EXE and ABA rats in the acute phase of the anorexic phenotype (PND42); **C** Example of full-size cropped immunoblots related to the expression levels of STAT3 (86 kDa) and β-actin (43 kDa) measured in the nuclear fraction of the dorsal Hippocampus of CTRL, FR, EXE and ABA rats in the acute phase of the anorexic phenotype (PND42).
1= controls, CTRL; 2= food restricted, FR; 3= exercise, EXE; 4= activity-based anorexia, ABA.

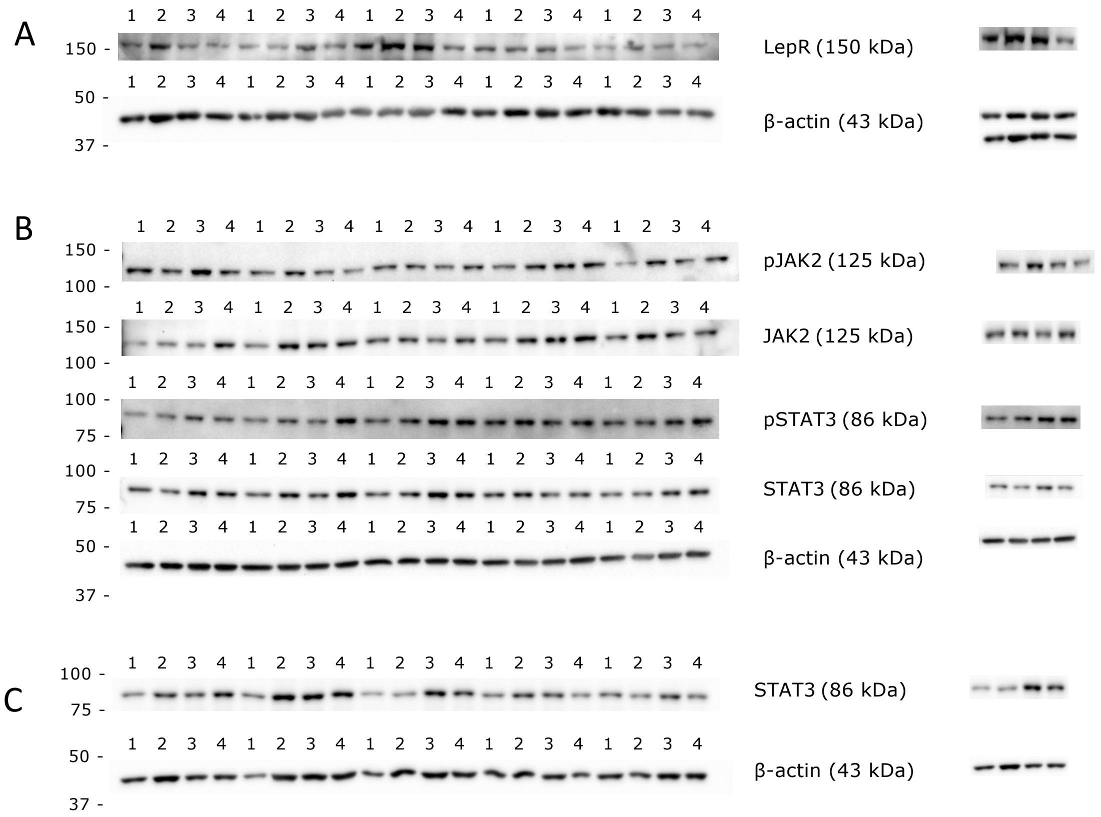

Supplementary figure S4. A Example of full-size cropped immunoblots related to the expression levels of LepR (150 kDa) and β-actin (43 kDa) measured in the whole post-synaptic density of the dorsal Hippocampus of CTRL, FR, EXE and ABA rats after the recovery phase of the anorexic phenotype (PND 49); **B** Example of full-size cropped immunoblots related to the expression levels of pJAK2 (125 kDa), JAK2 (125 kDa), pSTAT3 (86 kDa), STAT3 (86 kDa) and β-actin (43 kDa) measured in the cytosolic fraction of the dorsal Hippocampus of CTRL, FR, EXE and ABA rats after the recovery phase of the anorexic phenotype (PND 49); **C** Example of full-size cropped immunoblots related to the expression levels of STAT3 (86 kDa) and β-actin (43 kDa) measured in the nuclear fraction of the dorsal Hippocampus of CTRL, FR, EXE and ABA rats after the recovery phase of the anorexic phenotype (PND 49).
1= controls, CTRL; 2= food restricted, FR; 3= exercise, EXE; 4= activity-based anorexia, ABA.

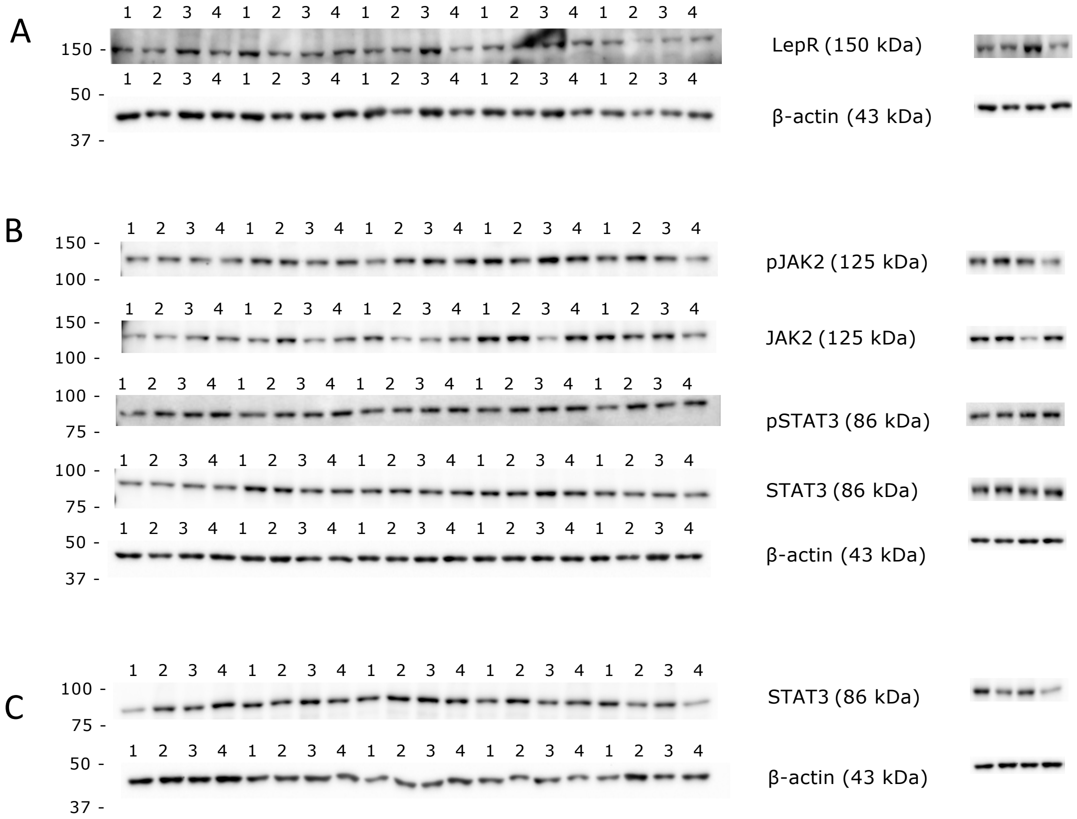

Supplementary Table S1. Two-way ANOVA analysis of leptin plasma levels (pg/ml) (A) in the acute phase of the phenotype (PND42) and (B) after a 7-day recovery period (PND49)

A

**Leptin plasma levels**	**Two-way ANOVA** (followed by Tuckey's post hoc test)	
**Acute phase**		
	F value	p value
Interaction	F (1, 16) = 7,716	0,0134
Exercise	F (1, 16) = 11,31	0,0040
Food restriction	F (1, 16) = 51,56	<0,0001

B

**Leptin plasma levels**	**Two-way ANOVA** (followed by Tuckey's post hoc test)	
**After recovery**		
	F value	p value
Interaction	F (1, 16) = 6,448	0,0219
Food restriction	F (1, 16) = 28,47	<0,0001

Supplementary Table S2. Table 1 Expression and phosphorylation levels of JAK2/STAT3 measured in the cytosolic (A) and (B) nuclear fraction of the ventral Hippocampus of CTRL, FR, EXE and ABA rats in the acute phase of the phenotype induction (left) and after a period of body weight recovery (right). Data are presented as the mean ± SEM.

*p<0.05, **p<0.01. ***p<0.001 vs. CTRL, #p<0.05, ##p<0.01, ###p<0.001 vs. FR, $p<0.05, $$p<0.01 vs. EXE. (Two-way ANOVA followed by Tukey's multiple comparisons test).

CTRL: controls (n=5/time point); FR: food restricted (n=5/time point); EXE: exercise (n=5/time point); ABA: activity-based anorexia (n=5/time point)

A

Cytosolic fraction	Acute phase				After weight recovery			
	**CTRL**	**FR**	**EXE**	**ABA**	**CTRL**	**FR**	**EXE**	**ABA**
**pJAK2**	100 ± 1	106 ± 9	117 ± 3	96 ± 4$	100 ± 3	135 ± 8	133 ± 7	142 ± 16
**JAK2**	100 ± 2	94 ± 10	92 ± 4	131 ± 10*/#/$$	100 ± 3	121 ± 4*	117 ± 3	94 ± 7##/$$
**pSTAT3**	100 ± 2	59 ± 5***	61 ± 3***	75 ± 4**/#	100 ± 7	91 ± 9	94 ± 4	124 ± 8#/$
**STAT3**	100 ± 5	83 ± 3	90 ± 8	115 ± 10#	100 ± 5	103 ± 4	103 ± 8	99 ± 5

B

Nuclear fraction	Acute phase				After weight recovery			
	**CTRL**	**FR**	**EXE**	**ABA**	**CTRL**	**FR**	**EXE**	**ABA**
**STAT3**	100 ± 6	81 ± 5	80 ± 3	71 ± 8	100 ± 5	105 ± 6	110 ± 4	137 ± 4***/###/$$

Supplementary Table S3. Expression and phosphorylation levels of JAK2/STAT3 measured in the cytosolic (A) and nuclear (B) fraction of the dorsal Hippocampus of CTRL, FR, EXE and ABA rats in the acute phase of the phenotype induction (left) and after a period of body weight recovery (right). Data are presented as the mean± SEM.

*p<0.05, **p<0.01 vs. CTRL, #p<0.05, ##p<0.01 vs. FR, $p<0.05, $$p<0.01 vs. EXE, @@p<0.01 vs. ABA. (Two-way ANOVA followed by Tukey's multiple comparisons test).

CTRL: controls (n=5/time point); FR: food restricted (n=5/time point); EXE: exercise (n=5/time point); ABA: activity-based anorexia (n=5/time point).

A

Cytosolic fraction	Acute phase				After weight recovery			
	**CTRL**	**FR**	**EXE**	**ABA**	**CTRL**	**FR**	**EXE**	**ABA**
**pJAK2**	100 ± 7	119 ± 4	116 ± 5	84 ± 7$^{\#\#/\$\$}$	100 ± 5	112 ± 8	106 ± 8	77 ± 3$^{\#\#/\$}$
**JAK2**	100 ± 9	113 ± 9	103 ± 12	132 ± 8	100 ± 9	105 ± 8	79 ± 11	101 ± 3
**pSTAT3**	100 ± 7	116 ± 6	111 ± 2	126 ± 3$^{**/\#\#/\$\$}$	100 ± 2	109 ± 5	104 ± 5	107 ± 5
**STAT3**	100 ± 1	120 ± 4	109 ± 5	124 ± 4	100 ± 5	97 ± 6	100 ± 5	98 ± 5

B

Nuclear fraction	Acute phase				After weight recovery			
	**CTRL**	**FR**	**EXE**	**ABA**	**CTRL**	**FR**	**EXE**	**ABA**
**STAT3**	100 ± 9	97 ± 7	136 ± 7$^{**/@@}$	99 ± 2	100 ± 5	103 ± 5	98 ± 2	79 ± 5$^{*/\$\$}$

Supplementary Table S4. Detailed F values, degrees of freedom and p values relative to two-way ANOVA analysis of protein levels evaluated in the ventral Hippocampus of CTRL, FR, EXE and ABA rats in the acute phase of the phenotype (A) and after a 7-day recovery period (B) and presented in figure 2 and 3 and table 1.

CTRL: controls; FR: food restricted; EXE: exercise; ABA: activity-based anorexia.

A

**Ventral Hippocampus**<br>**Acute phase**		**Two-way ANOVA**<br>**(followed by Tuckey's post hoc test)**	
Post-synaptic density		**F value**	**p value**
**Leptin Receptor**	Interaction	F (1, 16) = 31,08	<0,0001
	Exercise	F (1, 16) = 21,37	0,0003
	Food restriction	F (1, 16) = 8,452	0,0103
Cytosolic fraction			
**pJAK2 (Tyr1007/1008)**	Interaction	F (1, 16) = 6,817	0,0189
**JAK2**	Interaction	F (1, 16) = 9,453	0,0073
	Food restriction	F (1, 16) = 5,053	0,0390
**pJAK2/JAK2**	Interaction	F (1, 16) = 62,91	<0,0001
	Food restriction	F (1, 16) = 21,19	0,0003
**pSTAT3 (Tyr705)**	Interaction	F (1, 16) = 54,38	<0,0001
	Exercise	F (1, 16) = 9,258	0,0078
	Food restriction	F (1, 16) = 13,10	0,0023
**STAT3**	Interaction	F (1, 16) = 8,933	0,0087
Nuclear fraction			
**STAT3**	Exercise	F (1, 16) = 6,813	0,0189
	Food restriction	F (1, 16) = 5,480	0,0325
Cytosol to Nucleus translocation			
**STAT3**	Interaction	F (1, 16) = 8,158	0,0114
	Exercise	F (1, 16) = 27,26	<0,0001
	Food restriction	F (1, 16) = 11,87	0,0033

B

**After recovery**			
Post-synaptic density			
**Leptin Receptor**	Interaction	F (1, 16) = 5,825	0,0282
	Exercise	F (1, 16) = 5,572	0,0313
	Food restriction	F (1, 16) = 6,321	0,0230
Cytosolic fraction			
**pJAK2 (Tyr1007/1008)**	Food restriction	F (1, 16) = 5,070	0,0387
**JAK2**	Interaction	F (1, 16) = 24,75	0,0001
**pJAK2/JAK2**	Interaction	F (1, 16) = 4,667	0,0463
	Exercise	F (1, 16) = 19,17	0,0005
	Food restriction	F (1, 16) = 15,71	0,0011
**pSTAT3 (Tyr705)**	Interaction	F (1, 16) = 7,102	0,0169
**pSTAT3/STAT3**	Interaction	F (1, 16) = 9,109	0,0082
Nuclear fraction			
**STAT3**	Interaction	F (1, 16) = 5,375	0,0340
	Exercise	F (1, 16) = 20,12	0,0004
	Food restriction	F (1, 16) = 12,08	0,0031
Cytosol to Nucleus translocation			
**STAT3**	Interaction	F (1, 16) = 5,375	0,0340
	Exercise	F (1, 16) = 20,12	0,0004
	Food restriction	F (1, 16) = 12,08	0,0031

Supplementary Table S5. Detailed F values, degrees of freedom and p values relative to two-way ANOVA analysis of mRNA levels evaluated in the ventral Hippocampus of CTRL, FR, EXE and ABA rats in the acute phase of the phenotype (A) and after a 7-day recovery period (B) and presented in figure 3.

CTRL: controls; FR: food restricted; EXE: exercise; ABA: activity-based anorexia.

A

**Ventral Hippocampus Acute phase**		**Two-way ANOVA (followed by Tuckey's post hoc test)**	
mRNA levels		**F value**	**p value**
Socs3	Interaction	F (1, 16) = 34,09	<0,0001
Dnmt1	Interaction	F (1, 16) = 6,684	0,0199
	Food restriction	F (1, 16) =7,130	0,0168

B

**Ventral Hippocampus After recovery**		**Two-way ANOVA (followed by Tuckey's post hoc test)**	
mRNA levels		**F value**	**p value**
Socs3	Interaction	F (1, 15) = 8,868	0,0094
	Exercise	F (1, 15) = 7,637	0,0145
	Food restriction	F (1, 15) =9,230	0,0083
Dnmt1	Interaction	F (1, 16) = 15,65	0,0011
	Exercise	F (1, 16) = 61,23	<0,0001
	Food restriction	F (1, 16) = 32,03	<0,0001

Supplementary Table S6. Detailed F values, degrees of freedom and p values relative to two-way ANOVA analysis of protein levels evaluated in the dorsal Hippocampus of CTRL, FR, EXE and ABA rats in the acute phase of the phenotype (A) and after a 7-day recovery period (B) and presented in figure 4 and 5 and table 2.

CTRL: controls; FR: food restricted; EXE: exercise; ABA: activity-based anorexia.

A

**Dorsal Hippocampus Acute phase**		**Two-way ANOVA (followed by Tuckey's post hoc test)**	
Post-synaptic density		**F value**	**p value**
**Leptin Receptor**	Interaction	F (1, 16) = 17,83	0,0006
	Food restriction	F (1, 16) = 11,76	0,0034
Cytosolic fraction			
**pJAK2 (Tyr1007/1008)**	Interaction	F (1, 16) = 19,39	0,0004
**JAK2**	Food restriction	F (1, 16) = 4,753	0,0445
**pJAK2/JAK2**	Interaction	F (1, 16) = 4,621	0,0472
**pSTAT3 (Tyr705)**	Interaction	F (1, 16) = 5,169	0,0371
	Exercise	F (1, 16) = 9,348	0,0075
	Food restriction	F (1, 16) = 8,837	0,0090
**STAT3**	Exercise	F (1, 16) = 12,36	0,0029
	Food restriction	F (1, 16) = 7,951	0,0123
Nuclear fraction			
**STAT3**	Interaction	F (1, 16) = 6,689	0,0199
	Exercise	F (1, 16) = 8,556	0,0099
	Food restriction	F (1, 16) = 9,928	0,0062
Cytosol to Nucleus translocation			
**STAT3**	Interaction	F (1, 16) = 11,10	0,0042
	Food restriction	F (1, 16) = 32,11	<0,0001

B

**After recovery**			
Post-synaptic density			
**Leptin Receptor**	Interaction	F (1, 16) = 17,28	0,0007
	Exercise	F (1, 16) = 12,85	0,0025
	Food restriction	F (1, 16) = 62,88	<0,0001
Cytosolic fraction			
**pJAK2 (Tyr1007/1008)**	Interaction	F (1, 16) = 10,13	0,0058
	Exercise	F (1, 16) = 4,919	0,0414
**pJAK2/JAK2**	Interaction	F (1, 16) = 31,00	<0,0001
	Food restriction	F (1, 16) = 23,10	0,0002
Nuclear fraction			
**STAT3**	Interaction	F (1, 16) = 8,651	0,0096
	Food restriction	F (1, 16) = 13,00	0,0024
Cytosol to Nucleus translocation			
**STAT3**	Interaction	F (1, 16) = 20,55	0,0003
	Food restriction	F (1, 16) = 19,82	0,0004

Supplementary Table S7. Detailed F values, degrees of freedom and p values relative to two-way ANOVA analysis of mRNA levels evaluated in the dorsal Hippocampus of CTRL, FR, EXE and ABA rats in the acute phase of the phenotype (A) and after a 7-day recovery period (B) and presented in figure 5.

CTRL: controls; FR: food restricted; EXE: exercise; ABA: activity-based anorexia.

A

**Dorsal Hippocampus Acute phase**		**Two-way ANOVA (followed by Tuckey's post hoc test)**	
mRNA levels		**F value**	**p value**
Socs3	Exercise	F (1, 16) = 8,331	0,0507
Dnmt1	Interaction	F (1, 16) = 23,28	0,0002
	Exercise	F (1, 16) = 6,126	0,0249

B

**Dorsal Hippocampus After recovery**		**Two-way ANOVA (followed by Tuckey's post hoc test)**	
mRNA levels		**F value**	**p value**
Socs3	Exercise	F (1, 16) = 42,43	<0,0001
Dnmt1	Interaction	F (1, 16) = 14,34	0,0016
	Exercise	F (1, 16) = 15,92	0,0011
	Food restriction	F (1, 16) = 23,36	0,0002
